# Novel Drop‐Sampler for Simultaneous Collection of Stereo‐Video, Environmental DNA and Oceanographic Data

**DOI:** 10.1002/ece3.70705

**Published:** 2024-12-23

**Authors:** Samuel Thompson, Simon Jarman, Kingsley Griffin, Claude Spencer, Gabrielle Cummins, Julian Partridge, Tim Langlois

**Affiliations:** ^1^ School of Biological Sciences The University of Western Australia Perth WA Australia; ^2^ UWA Oceans Institute The University of Western Australia Perth WA Australia; ^3^ School of Molecular and Life Sciences Curtin University Perth WA Australia

**Keywords:** biodiversity, BRUVs, drop‐camera, eDNA, fish ecology, metabarcoding, sampling method, stereo‐video

## Abstract

There is an increasing interest in environmental DNA (eDNA) as a method to survey marine biota, enhancing traditional survey methods, and a need to ground truth eDNA‐based interpretations with visual surveys to understand biases in both the eDNA and visual datasets. We designed and tested a rapidly deployable, robust method pairing water sampling for eDNA collection and stereo‐video imagery, comparing inferred fish assemblages with interspersed baited remote underwater video (stereo‐BRUV) samples. The system is capable of rapidly collecting simultaneous wide‐field stereo‐video imagery, oceanographic measurements and multiple water samples across a range of habitats and depths (up to 600 m). A platform demonstration was conducted in a no‐take National Park Zone of the Ningaloo Marine Park, Western Australia, with samples being collected whilst the system is resting on the seafloor. Combining simultaneous visual survey data with eDNA species estimates increased the total diversity of the fish assemblage by ca. 6.5% over eDNA estimates alone, whilst the analysis of the assemblage composition sampled by each method revealed significant differences. The platform demonstration highlights the biases of each sampling method and their complementarity to one another. We suggest that these biases will be better understood by advancements that allow eDNA metabarcoding to discriminate the abundance and life stage of marine biota. Furthermore, investigation of the relationship between eDNA metabarcoding data and concomitant imagery‐derived length, age and habitat data is needed.

## Graphical abstract

We designed and tested a novel, rapidly deployable, robust method for marine fish assemblages, pairing water sampling for eDNA collection and stereo‐video imagery, whilst comparing inferred fish assemblages with interspersed baited remote underwater video (stereo‐BRUV) samples.

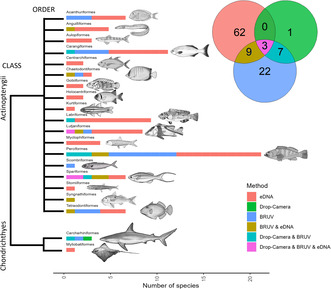

## 1 Introduction

The diversity of marine life in the world's oceans is changing. The growing human population is exacting an increasing cumulative impact on the marine environment through climate change, overfishing, land‐based pollution and offshore industries such as energy and maritime shipping, leading to a rapid decline in populations and species across almost every marine ecosystem on Earth (Valdés et al. 2009; Halpern et al. 2019). Marine benthic habitats in particular support a range of important ecosystem services, including fisheries productivity (Kritzer et al. 2016) and ecosystem functions including biogeochemical processes such as nutrient cycling and carbon sequestration (Griffiths et al. 2017; Atwood et al. 2020) and the provision of shelter and nursery areas for fauna (Nagelkerken et al. 2002; Barbier et al. 2017). The ongoing degradation of these habitats, particularly biogenic habitats in coastal zones (Waycott et al. 2009; Beck et al. 2011; Sunday et al. 2016), largely through the use of bottom‐contacting mobile fishing gear (Amoroso et al. 2018), often leads to lower abundances (biomasses) and declines in species richness (Airoldi, Balata, and Beck 2008). Furthermore, as new technologies and rising demand have caused the intensity and effects of fishing and industrialisation to increase over time, there has been a rapid global decline in fisheries targeting large predatory finfish (Pontecorvo and Schrank 2012; Ritchie and Roser 2021), resulting in fishers increasingly targeting smaller pelagic species and bottom‐dwelling invertebrates (Essington, Beaudreau, and Wiedenmann 2006; Estes et al. 2011). In many cases, this “fishing down the food web” can result in dramatic ecosystem shifts to states that are both ecologically and economically undesirable, being difficult and expensive to reverse (Howarth et al. 2014). Construction of offshore wind facilities (Bilgili and Alphan 2022) and the issue of exploration contracts for deep‐sea mining (“ISA” 2022) are also increasing, adding further potential pressures to marine ecosystems and increasing competition for resources and space.

In response to these challenges, policy makers have implemented ecosystem‐based management (EBM) programmes, essentially reversing the order of management priorities to prioritise ecosystem functions over target species (Pikitch et al. 2004), and designated thousands of Marine Protected Areas (MPAs) in recent years, with a global total of 18,200 MPAs currently covering approximately 8% of the world's oceans (UNEP‐WCMC and IUCN 2024). When properly designed and managed, MPAs serve as valuable tools for reducing human impacts on marine environments (Edgar et al. 2014; Giakoumi et al. 2018; Sciberras et al. 2018). They enhance biological communities both within and outside their boundaries (Di Lorenzo, Claudet, and Guidetti 2016; Sala and Giakoumi 2018), improve ecosystem functions, and bolster natural capital assets (Leenhardt et al. 2015; Rasheed 2020). However, numerous research works have discussed the actual level of safeguarding provided by MPAs (Edgar et al. 2014; Strain et al. 2019), highlighting the risk of “paper parks” that do not effectively achieve their intended objectives (Rife, Erisman, and Sanchez 2013). Despite some pitfalls of MPAs, particularly when policies are not enforced, EBM is still recognised as best practice for managing multiple ocean uses and associated sectors, explicitly addressing the trade‐offs among them (Curtin and Prellezo 2010; Link and Browman 2017). Marine EBM however, is, by its very nature difficult and complex to implement, requiring a broad range of survey data across a suite of biological indicators (Zador et al. 2017; Petit‐Marty, Casas, and Saborido‐Rey 2023).

Baited Remote Underwater stereo‐Video (stereo‐BRUVs) is a widely implemented, non‐destructive sampling technique capable of providing relative abundance by the size class for bony fishes (Bosch et al. 2022), elasmobranchs (i.e., sharks, rays and skates; Goetze et al. 2018) and of characterising the recovery of benthic habitats from trawling (Langlois et al. 2021). These systems comprise a pair of high‐definition video cameras having optical axes converging at *ca*. 7° to provide an overlapping field of view (Langlois et al. 2010; Watson et al. 2010), recording relative abundance (Watson et al. 2005; Harvey et al. 2007), by size–class information (Langlois et al. 2015; Bornt et al. 2015), whilst allowing the sample unit to be standardised across varying visibility (Broad et al. 2023). Stereo‐BRUVs can sample beyond the depth limits of SCUBA, including the deep ocean (Marouchos et al. 2011), are fishery‐independent and suited to sampling a diverse range of fish assemblages, including rocky habitats, with a high sampling efficiency in the field.

Stereo‐BRUVs however, are time‐consuming and expensive to analyse, requiring extensive taxonomic expertise, which makes them difficult to scale up across large ecosystems (Noble‐James et al. 2023). They also have inherent, previously characterised biases in their sampling (Langlois et al. 2015), such as difficulties in determining the water volume sampled due to variables associated with the dispersion of attractants from the bait, conservative relative abundance estimation (Farnsworth et al. 2007), reliance on acceptable underwater visibility, and an inability to detect more cryptic reef‐associated species (Watson et al. 2005), or identify very small (including larval) animals.

Environmental DNA (eDNA) metabarcoding has enabled the genetic detection and profiling of a wide range of biota present in environmental samples (e.g., water, scat, soil, etc.) and has proven value in the measurement of species presence, diet and the detection of invasive species (West et al. 2021; Deagle et al. 2007; Pearman et al. 2021). It is well suited to the marine environment because DNA is detectable for up to ~48 h in nearshore environments (Collins et al. 2018). It is particularly useful for detecting cryptic species, as it circumvents the constraints associated with taxonomic identification in visual approaches, increasing taxonomic resolution (Deiner et al. 2017). Furthermore, the use of multiple genetic markers allows the sampling of a broader spectrum of diversity (compared to visual methods) from across the tree of life (Stat et al. 2017). eDNA metabarcoding is also suited to detecting rare species that would be difficult to detect by traditional means but not for determining species abundance, body‐size condition or sex ratios and thus is likely to be complementary to established methods such as stereo‐BRUVs studies (Stat et al. 2019). Researchers typically use filtration to collect eDNA from water samples (Takahashi et al. 2023), though due to its time‐consuming nature and the tendency for membranes to become blocked by particulates in turbid waters, researchers have more recently started exploring alternative approaches. Examples include in situ remotely deployed instruments that automate filtering (McQuillan and Robidart 2017), high‐volume sampling using tow nets (Schabacker et al. 2020), passive collection using membranes submerged at the site (Kirtane, Atkinson, and Sassoubre 2020; Bessey et al. 2021), in vitro passive sampling (whereby a adsorbent material is placed inside a discrete water sample, thus allowing the control of the sampling volume: Thompson et al. unpublished data) or by recovering eDNA from natural filter feeders, e.g., sponges (Mariani et al. 2019) and mussels (Jeunen et al. 2023).

eDNA metabarcoding methodology is, however, still developing, and the ecological interpretation of information derived from eDNA collection often benefits from ground truthing. To this end, it is useful to collect paired or interspersed imagery or visual data in order to ground truth and start to understand biases in both the novel eDNA and established imagery‐based methods such as stereo‐BRUVs. There is also a need for standardisation of collection and processing of samples when conducting eDNA surveys at varying spatial and temporal scales (Wort et al. 2022). Furthermore, as aquatic DNA is collected in complex and variable hydrological systems, eDNA transport is a key consideration for inferring the presence and spatial distribution of species. This includes horizontal and vertical transport, retention in benthic sustrate and the probability of resuspension into the water column (McNair and Newbold 2012), the rates of which may be confounded with the residency time of eDNA within the environment prior to decay. Gathering environmental variables, including temperature, salinity, turbidity, microbial growth (chlorophyll α) and nutrient levels alongside metabarcoding data can facilitate the construction of process‐based models of eDNA transport and degradation so that environmentally driven variation can be assessed (Harrison, Sunday, and Rogers 2019). Such models are important for understanding the spatial and temporal resolution of eDNA when assessing the genetic material captured within a seawater sample.

Spatially balanced survey designs can increase the sampling efficiency by evenly distributing samples in space and across the ranges of covariates of interest (e.g., depth and habitat types) (Robertson et al. 2013). Typical platforms for collecting benthic imagery, i.e., divers, towed video, remotely operated vehicles (ROV) and autonomous underwater vehicles (AUV) have logistical constraints that result in them generally being deployed along transects or in discrete patches or mosaics (Sheehan et al. 2016). eDNA is often collected at discrete sites of interest with known differences in habitats; however, current methods of collecting seawater samples for genetic analysis such as surface water samples and Niskin bottle with messenger are not amenable to rapid point sampling across a variety of depths, and larger Niskin rosette systems typically require expensive vessels with specialised infrastructure including advanced winches and cables for lowering, retrieving and communicating with the Niskin rosette system.

To address the above issues, we have developed and tested a modified version of the benthic observational sampling system (BOSS), the Drop‐Camera system (Langlois et al. unpublished data), which has four pairs of cameras in a stereo‐video configuration and is amenable to spatial point sampling. The system is cuboid in shape with structural bars to protect the internal components, allowing the system to rest on the seafloor without risking damage to internal components. We added the capacity to collect discrete water samples at chosen depth(s) using Niskin rosette equipment (processed shipboard for eDNA metabarcoding) and a CTD for collecting oceanographic physical covariates including temperature, salinity, dissolved oxygen, fluorescence (chlorophyll‐α) and turbidity. The modified system, hereafter referred to as the “OceanBOSS” (Oceanographic and Benthic Observational Sampling System) can be rapidly deployed and retrieved from a variety of vessels into water depths up to 600 m, is self‐righting on the seabed, and a single deployment in 30 m of water takes 8 min with a 5 min bottom time. This tool is suited to the collection of widespread georeferenced point samples in shelf waters, collecting stereo‐video imagery, water samples and environmental covariates in one unified system, enabling rapid and cost‐effective sampling of broad areas using spatially balanced sampling designs, and can also produce data required for benthic habitat coverage predictions (Langlois et al. unpublished data).

We investigate the potential of this modified system to collect demersal fish assemblage information by using metabarcoding of eDNA collected from water samples (collected whilst the system rests on the seafloor) and images from the wide‐field camera system. We compare these assemblage estimates to those derived from interspersed stereo‐BRUV imagery collected on separate days of the same voyage, with the aim of understanding the strengths and biases of each sampling method.

## 2 Methods

### 2.1 OceanBOSS Design

**FIGURE 1:**
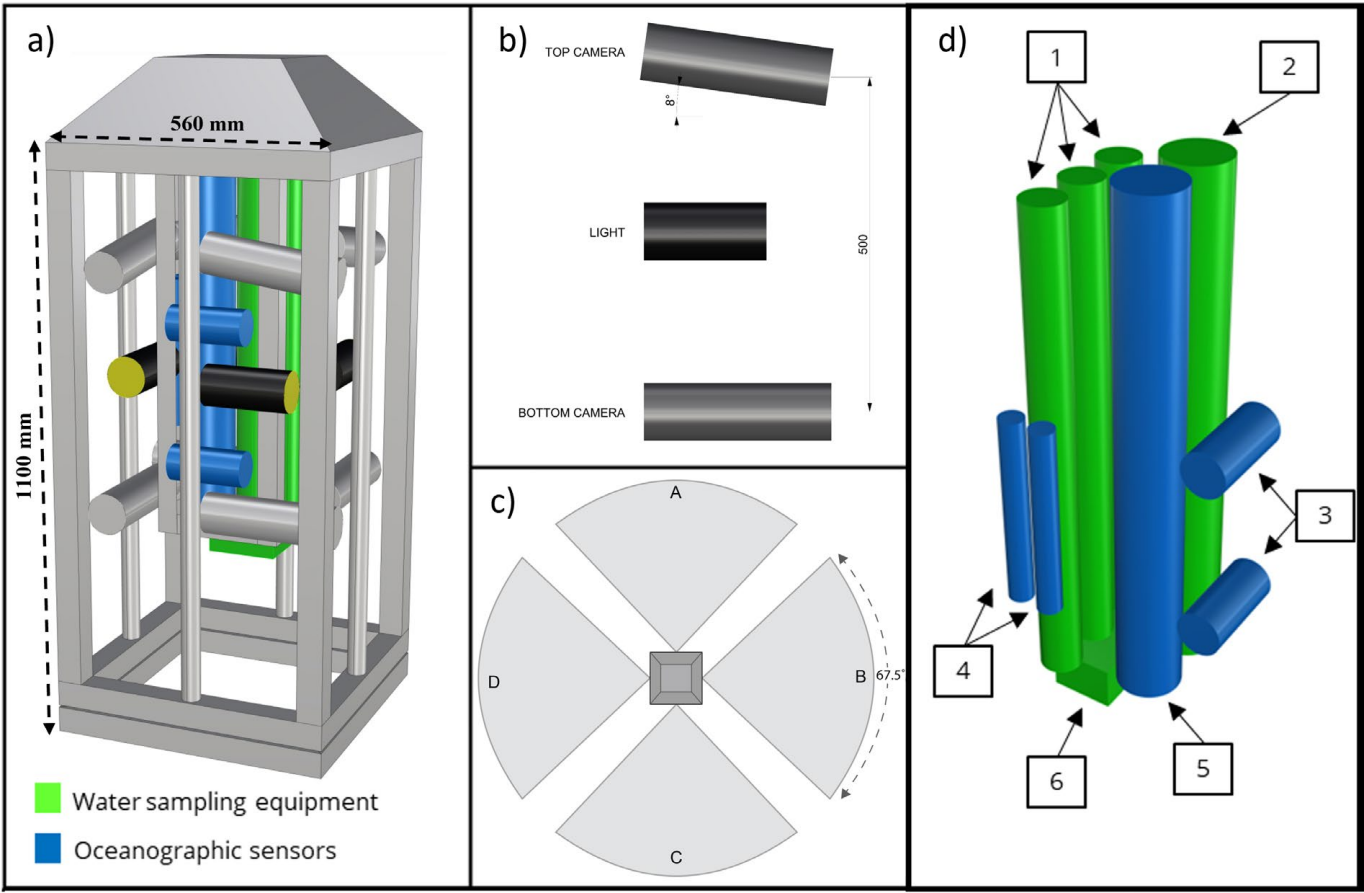
Wide‐field Drop‐Camera and the water sampler “OceanBOSS” system design. (a) Technical model of the Drop‐Camera system showing camera housings (grey) and lights (black). (b) Specifications of the camera separation and angle of convergence. (c) Overhead field of view highlighting the 270° field of view. (d) Technical model of the internal hardware: 1: Niskin bottles, 2: Seabird SBE55 3: Chelsea Technologies MiniTracka II Fluorometer and Nephelometer. 4: Seabird SBE5T and SBE43. 5: Seabird SBE19plus. 6: Seabird SBE55 rosette latch housing.

The OceanBOSS system features a sturdy aluminium frame to secure and protect its internal equipment, which includes eight stereo‐video cameras with associated LED lights, Niskin bottles for water sample collection, a CTD (Conductivity, Temperature and Depth) sensor, and additional sensors to measure dissolved oxygen, Chl‐*a* and turbidity (Figure 1d). A removable weight (16 kg) attached to the bottom, along with syntactic foam packed into the top of the frame allow the OceanBOSS to maintain its vertical orientation during its descent to the seabed and in the presence of seafloor currents. The OceanBOSS can be deployed with floats on the rope, removing the need for a permanent tether to the boat, and all components are pressure‐rated to at least 600 m depth.

### 2.2 Sampler Operation

#### 2.2.1 Deployment and Retrieval

**FIGURE 2:**
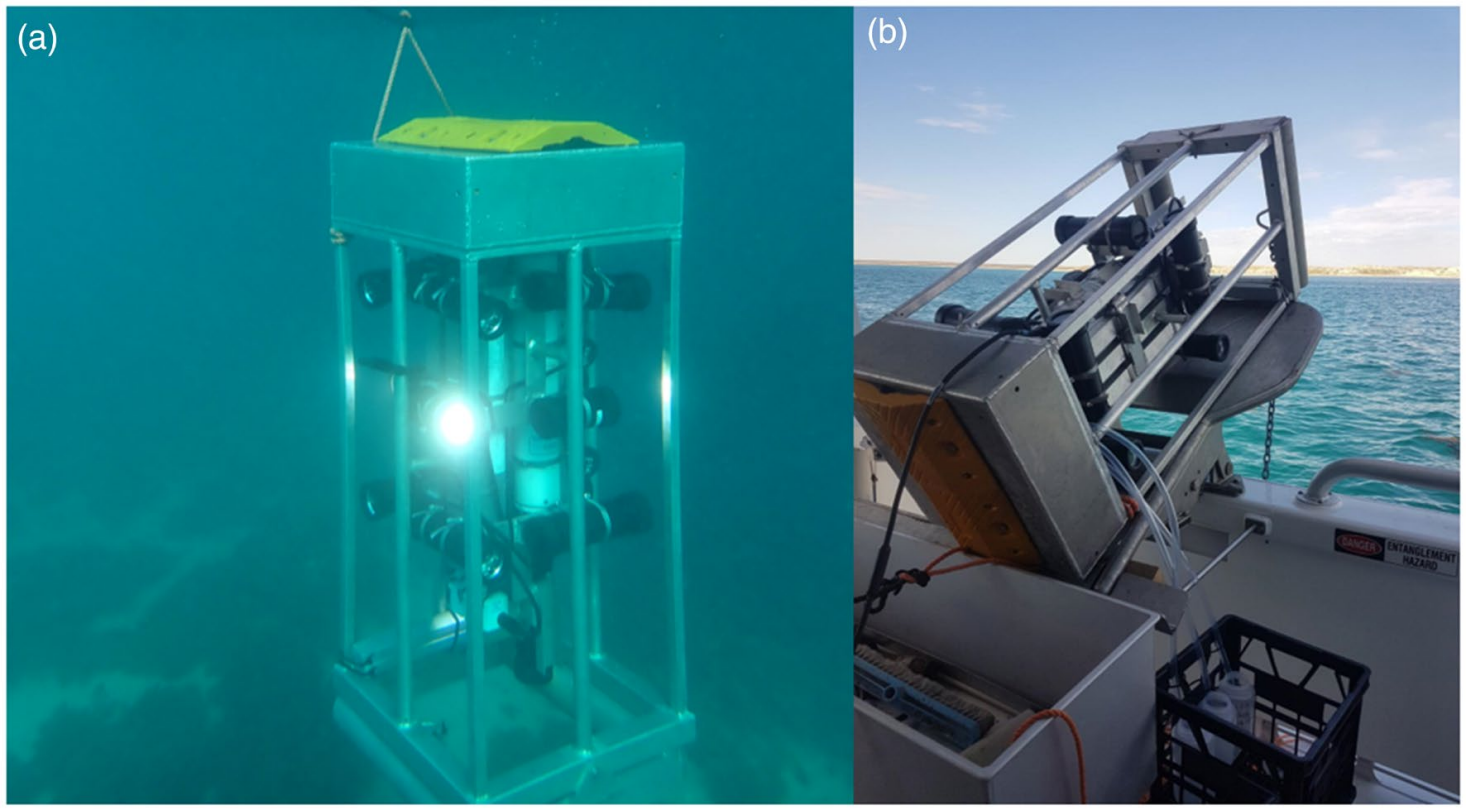
Panel a: The sampler during its descent to the seabed. Panel b shows the system configuration after retrieval and water extraction into bottles.

The sturdy rectangular aluminium frame is well‐suited for deployment and retrieval from vessels fitted with fish‐ or crustacean‐trap retrieval equipment such as a davit arms or pot tippers or hydraulic pot winches. Its significant weight (65 kg) and buoyancy ensure a static, upright orientation (Figure 2a), even in deeper or fast‐flowing waters. These vessels are typically suited to the local sea conditions, and fisher‐skippers often provide valuable logistical advice and local knowledge.

Using a hydraulic winch, the sampler can be lowered in a controlled manner to a given depth and to avoid impacting the seabed with excessive force if sampling on the ocean floor. Markings or varying colours of deployment rope can help in tracking the depth of the OceanBOSS during its descent. The OceanBOSS should be deployed for a standard duration among all samples in a survey. We recommend deployments of 3–5 min bottom time, which if landing on the seafloor will allow any disturbed sediment time to settle, allowing clear footage of the surrounding habitat and fish, and to reduce the sediment loading in the water samples, which can inhibit PCR (Sidstedt, Rådström, and Hedman 2020). Shorter deployments may be sufficient for areas with limited sediment, and the ideal deployment length for optimal fish imagery and water samples can be determined in future studies. The Niskin bottles can also be set close after a given amount of time, which should be varied depending on the depth/descent rate at the sampling site, resettlement time of sediment and the deployment duration.

### 2.3 Sampling Design

**FIGURE 3:**
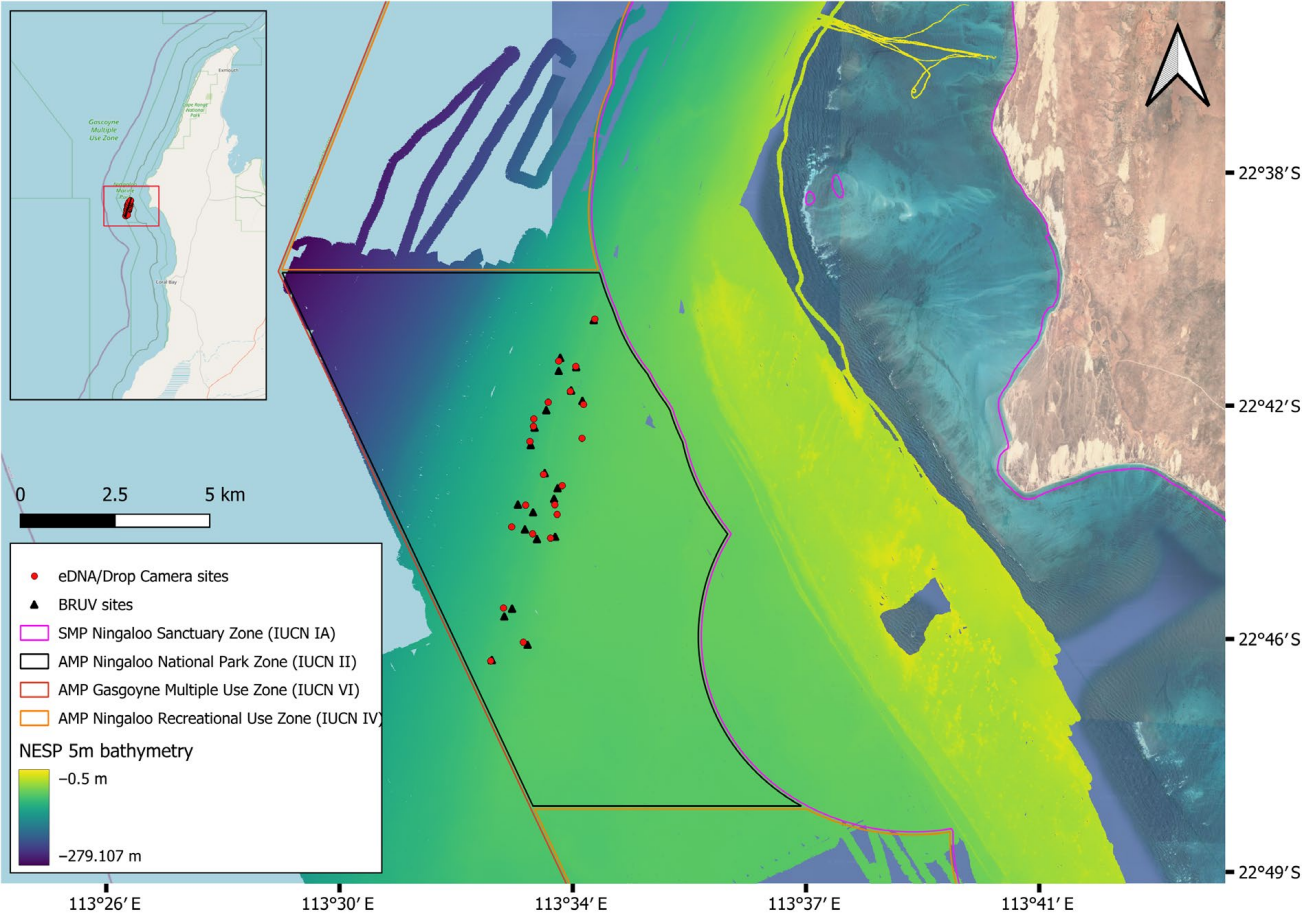
Map of the sampling area at Point Cloates. Red points indicate drop‐sampler sites, whilst black triangles indicate nearby BRUV sites. State marine park (SMP) and Commonwealth marine park (AMP) boundary zones are overlaid on a 5 m resolution bathymetric dataset from the National Environmental Science Program (NESP; Aston et al. 2022).

Concurrent water samples, stereo‐videography and CTD profiles were collected from 21 sites (Table S1) using the OceanBOSS drop‐sampler system at Point Cloates (Figure 3), in the Ningaloo region of Western Australia from the 21st to the 26th of May 2022 from the vessel Keshi Mer II. A bottom recording time of 5 min was used, whilst site depths varied between 72.1 and 94.5 m and covered a total area of 24.8 km^2^. To facilitate comparison with well‐established methods, 21 BRUV samples (Table S1) were also taken during this period from nearby locations no further than 280 m from the nearest drop‐sampler site on separate days to eDNA sampling, to reduce the risk of contamination from bait (Sardinops spp.).

#### 2.3.1 Water Sample Collection

Three 1.2 L seawater replicates were collected at each site, using Niskin bottles (General Oceanics model 1010; General Oceanics, Florida, USA), resulting in a total of 63 eDNA samples. The Niskin bottles were set to close at an elapsed time of 2, 3, and 4 min after sampler deployment, capturing water whilst the sampler sits on the seafloor. Seawater samples were transferred from the Niskin bottles to sterilised 1 L Nalgene bottles and immediately stored on ice. Each sample was individually filtered across a Pall GN‐6 Metricel 0.45 μm 47 mm membrane using a Pall Sentino Microbiology pump (Pall Corporation, Port Washington, USA), within 5 h of collection. After the filtration of water samples from each site, 10% bleach (sodium hypochlorite, prepared daily), followed by deionised water (prepared for each site) was used to rinse the filtration equipment, and a sample of deionised water (1 L) was included as a negative control before the filtration of water from the next site. A sample of the 10% bleach solution used to clean the filtration equipment was also included as a filtration control at the end of each day. These filtration controls serve to detect any potential cross‐contamination in water filtered from successive sites and during handling post‐filtration. After filtration, filter membranes were immediately placed in 540 μL buffer ATL (Qiagen; Venlo, the Netherlands) and stored at 4°C prior to their transportation to the Ecological Genetics Laboratories within the Indian Ocean Marine Research Centre (IOMRC).

#### 2.3.2 Oceanographic Data Collection

Oceanographic data were collected using an SBE19plus CTD (Sea‐Bird Scientific, Washington, USA), and additional sensors include an in situ fluorometer Chelsea MiniTracka II (Chelsea technologies, Surrey, UK), a Chelsea MiniTracka II nephelometer (Chelsea technologies, Surrey, UK) and an SBE43 dissolved oxygen (DO) sensor (Sea‐Bird Scientific, Washington, USA). A SBE5T DC pump (Sea‐Bird Scientific, Washington, USA) provides flow to the DO sensor and subsequently the conductivity cell through a 10 mm vinyl pipe. SBE19plus is connected to SBE55, and configuration and data storage are controlled through the same interface and program as SBE55. The CTD is configured in profiling mode, collecting four data points per second, and a magnetic switch is used to control data acquisition. CTD profiles can be stored in internal memory until uploaded.

Raw CTD data files (.hex) were processed using the program SeaSave (Version 7.26.7.121; Sea‐Bird Scientific, Washington, USA), discarding the upcast profile. Calibration data were applied from the last system calibration conducted by the manufacturer. Temperature data are expressed in °C (ITS‐90), salinity in practical salinity units (PSU), oxygen in % saturation, fluorescence in chlorophyll‐α μg/μL and optical backscatter (turbidity) in formazin turbidity units (FTU). Processed files were exported into R, where anomalous data points from the surface (0 m or higher) and for sediment disturbance after contact with the seafloor (datapoints recorded after the maximum depth value) were removed prior to analysis. Minimum, maximum and mean values for each measurement were then calculated from each profile and tested against depth (Figures S2–S4).

#### 2.3.3 Camera and Photogrammetry

Camera specifications can influence the accuracy of taxonomic identifications, and stereo‐measurements require careful adherence to camera alignment and calibration protocols. In our system, we record video using Sony FDR‐X3000 cameras, set to record at 1920 × 1080 pixels, at the frame rate of 60 frames per second and a ‘medium’ horizontal field of view setting (~67.5°).

Video files are written to removable internal memory (256 Gb SD cards), removing the requirement for a fibre optic cable to a separate surface or subsea recorder. Cameras are mounted on the frame with a 500 mm vertical separation and 8° downward angle on the top camera to maximise an overlapping field of view. Each camera is secured in a custom‐built housing and mounted to steel channels inside the frame. These channels are contained in a large internal structure (Figure 1a) which is not directly connected to the corners of the frame, which are subjected to greatest forces during deployment and retrieval. This protects camera calibration, necessary for accurate size measurements by minimising the movement of cameras during deployments and ensures camera alignment between deployments. The stereo‐video systems are calibrated in a pool prior to and post deployment in the field. Further information on the calibration of underwater stereo‐video camera systems can be found in the study of Harvey and Shortis (1998). Illumination for deeper deployments is provided by a single white LED array light (CREE XPGBWT, 9 w, 500 lx at 1 m) mounted between the camera pairs for each direction. To ensure that the images from each camera can be effectively composited to be viewed simultaneously, the camera systems require synchronisation which is achieved using an LED resyncing diode (see Langlois et al. unpublished data).

Analysis of video footage was facilitated through EventMeasure Stereo software (SeaGIS; Bacchus Marsh, Australia). Individual fish were identified to the lowest taxonomic level achievable. Individuals which could not be confidently or consistently identified to species level were identified to genus or family level where possible following Langlois et al. (2020).

### 2.4 Laboratory Processing

DNA was extracted from the filter membranes within 4 weeks of collection using a DNeasy blood and tissue kit (Qiagen; Venlo, Netherlands) with the following modifications: 540 μL Buffer ATL (added in the sampling step above) and 60 μL proteinase K added during the digestion phase. The second elution step was also completed as per the manufacturer's protocol. Negative controls, containing no sample (or filters), were extracted and processed alongside samples in order to detect any cross‐contamination during the extraction process. Three PCR assays, herein referred to as 16S‐Lutjanidae (Appendix S1), 16S‐fish (Deagle et al. 2007; Berry et al. 2017) and 16S‐Fish_SynShort (Nester et al. 2020) were incorporated into our eDNA metabarcoding workflow (Table 1).

**TABLE 1:** PCR assay information for marine eDNA metabarcoding at Point Cloates.

PCR assay	Primer name	Oligonucleotide sequence (5′–3′)	Internal region size (bp)	Annealing temperature (°C)	References
16S‐Lutjanidae	16S_Lutjanidae_F	ACCCGTCTCTGTGGCAAAAGAGTGGG	~144	65	This study
	16S_Lutjanidae_R	GGCTCTCTCAATCAGTTTCCCCCAT			
16S‐Fish	16SF/D	GACCCTATGGAGCTTTAGAC	~200	54	Berry et al. (2017)
	16S2R‐degenerate	CGCTGTTATCCCTADRGTAACT			Deagle et al. (2007)
16S‐Fish_SynShort	16S_FishSyn_Short_F	GACGAGAAGACCCTGTGGAGC	~80	55	Nester et al. (2020)
	16S_FishSyn_Short_R	CCGYGGTCGCCCCAAC			

*Note:* Three assays, 16S‐Lutjanidae, 16S‐Fish and 16S‐FishSynShort, targeting the mitochondrial 16S rDNA gene region were applied to all collected seawater samples. In the primer name, “F” refers to the forward primer and “R” to the reverse primer.

Quantitative PCR (qPCR) amplification was performed in two steps using a QuantStudio 3 Real‐Time PCR System (Applied Biosystems). The first PCR reaction consisted of primers (Table 1) containing the Illumina sequencing adaptor and a primer sequence from each respective assay (Figure S1). The second reaction consisted of Illumina flow cell adaptor sequences (P5 and P7), a unique index (10 bp in length) and a portion of the Illumina sequencing adaptor (Figure S1). All qPCR reactions were prepared in a dedicated DNA‐free ultra‐clean laboratory facility at the Indian Ocean Marine Research Centre, Western Australia. Each first^t^ step qPCR reaction was carried out in triplicate 10 μL reactions containing: 5 μL PowerUp SYBR Green Master Mix (Applied Biosystems, MA, USA), 0.1 μL BSA (Thermo Fisher Scientific, MA, USA), 0.25 μL of each forward and reverse primers at 10 μM (Integrated DNA Technologies, Australia) and 4.4 μL of eDNA template.

Triplicate reactions from the same template were combined and tagged with a second PCR reaction to add sequencing adaptors and dual indexes in a 10 μL reaction containing 5 μL PowerUp SYBR Green Master Mix (Applied Biosystems), 1 μL each of forward and reverse primers at 10 μM (Integrated DNA Technologies), 1 μL of pooled first^t^ step amplicons and 2 μL of UltraPure DNase/RNase‐free distilled water (Thermo Fisher Scientific). Tagged amplicons were then pooled into five libraries based on the ranges of endpoint fluorescence values. Each library was subjected to a double‐sided clean‐up (Quail, Swerdlow, and Turner 2009) using AmpureXP (Beckman Coulter, CA, USA) magnetic beads, at a bead:sample ratio of ×0.9, ×1 and ×1.1 for 16S‐Fish_SynShort, 16S‐Lutjanidae and 16S‐Fish libraries, respectively, for the left‐side clean‐up and a ratio of 1.8× for the right side. Libraries were quantified using a TapeStation 4150 with a D1000 ScreenTape (Agilent Technologies, CA, USA) and a Qubit 4.0 fluorometer (Invitrogen, Carlsbad, USA) and pooled into a final library in an equimolar fashion. 10 pM of the final library was loaded onto a 300 cycle MiSeq V2 standard flow cell on an Illumina MiSeq platform and unidirectionally sequenced (Illumina, San Diego, USA) at Genomics WA in Perth, Western Australia.

### 2.5 Bioinformatics and Taxonomic Assignments

Reads were demultiplexed, merged and filtered using a combination of bcl2fastq v2.20 (Illumina) and DADA2 v1.28.0 (Callahan et al. 2016) software. Quality filtering parameters included a maximum error of two and a minimum read length of 60, 100 and 150 bp for 16S‐Lutjanidae, 16S‐Fish and 16S‐Fish_SynShort amplicons, respectively, and the removal of chimeras. Quality‐filtered de‐replicated sequences were then queried against the National Centre for Biotechnology Information's (NCBI) GenBank nt nucleotide database (accessed in 2022; Benson et al. 2005) using BLAST+ (Camacho et al. 2009). Taxonomic assignments were made using a lowest common ancestor (LCA) script in eDNAflow (Mousavi‐Derazmahalleh et al. 2021), with sequences assigned to a species‐level if there was ≥ 98% sequence identity (from BLAST alignments) across the entire length of the amplicon to a reference barcode and if sequences from at least one other species within the same genus (if another species exists) were available and ≤ 98% identical.

### 2.6 Data Cleaning

The three metabarcoding assays ran across 63 seawater eDNA extracts (and 29 control samples) yielded a total of 5,164,800 sequencing reads. The mean (±SD) number of 16S rDNA sequences per replicate sample was 60,622.67 (±24,033.21) based on 3,819,228 reads which passed quality filtering. Two seawater replicates and all control samples returned no valid sequences and were removed from the analysis.

For each method, taxa unresolved at a species level (Table S3), taxa without occurrence records from the Atlas of Living Australia between Shark Bay and Port Hedland, Western Australia (see Belbin et al. 2021) or taxa not belonging to *Actinoperygii* or *Chondrichthyes* families were removed prior to analysis. Furthermore, all sequences assigned to *Sardinops* and *Engraulis* genera were also removed, despite being found locally, as we could not rule out contamination from bait. For the visual methods, taxa without reference sequences for at least one of our regions of interest were also removed prior to analysis as these taxa would not be detectable with metabarcoding (Table S3).

### 2.7 Statistical Analyses

The presence or absence of fish species based on eDNA, Drop‐Camera and BRUVs was determined for each site. Univariate analyses were performed using R v4.3.0 (R Core Team 2023), and rarefaction/extrapolation curves, along with sample coverage values were generated using the iNEXT package (Hsieh, Ma, and Chao 2016). PRIMER v. 7 (Clarke and Gorley 2015) was used to compare the effects of the method and site on taxonomic composition. Data were transformed into the presence–absence format, and a modified Gower resemblance matrix was created with a log base 2 scale. A permutational multivariate analysis of variance (PERMANOVA) with factors method (fixed; BRUV, Drop‐Camera, eDNA) and site (random) was performed using the PERMANOVA + add on in PRIMER v. 7 (Anderson, Gorley, and Clarke 2008). All PERMANOVA tests including pairwise comparisons were conducted using unrestricted permutation of raw data and 9999 permutations.

## 3 Results

**FIGURE 4:**
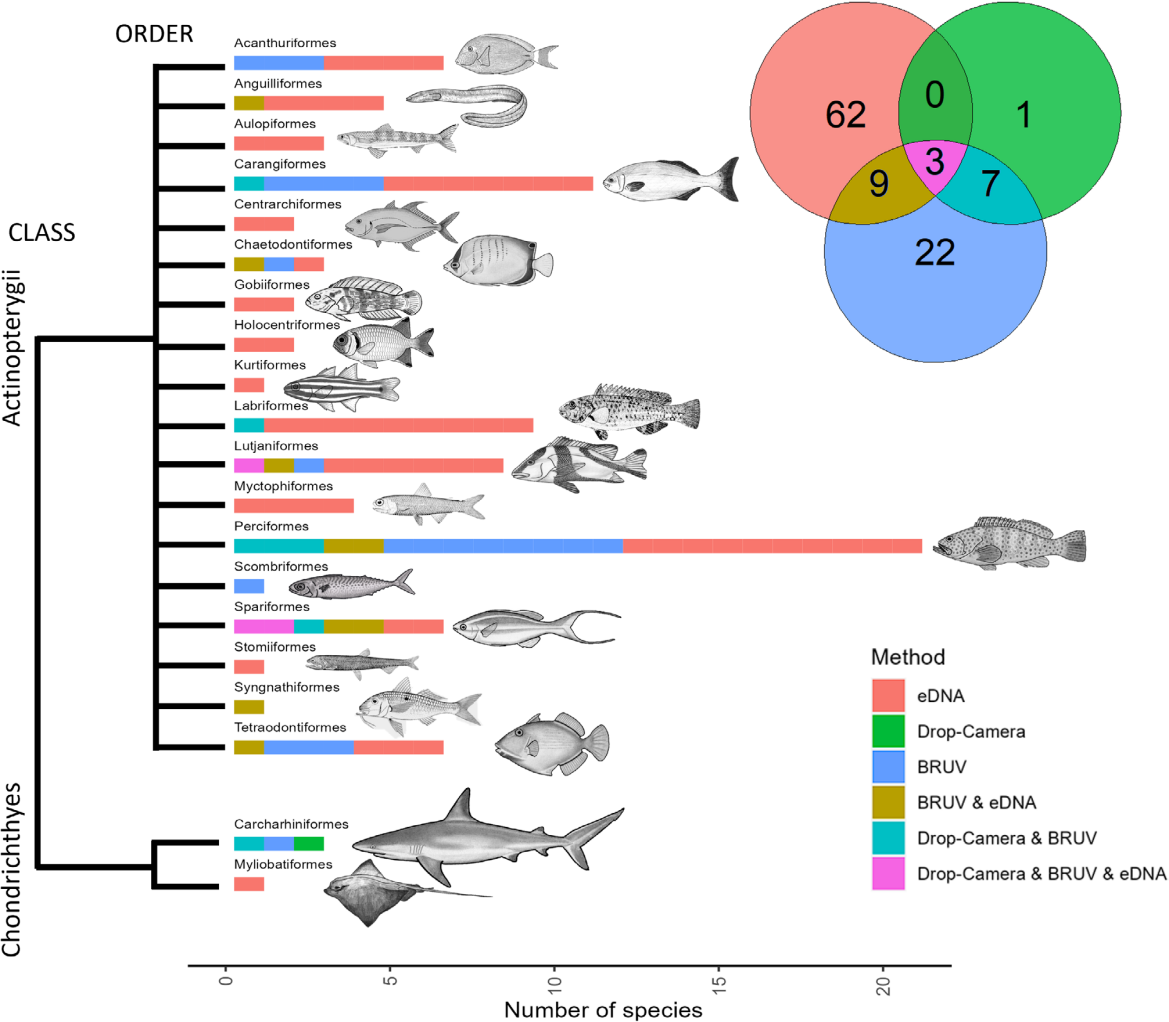
Number of species detected with each method, listed by class and order. The Venn diagram shows the number of species identified using methods in combination.

A total of 104 fish species from 20 orders and 2 classes (Actinopterygii and Chondrichthyes) were recorded across the sampling methods (OceanBOSS, eDNA and Drop‐Camera; and interspersed stereo‐BRUV survey) (Figure 4). The observed species richness per site was higher in eDNA samples (mean = 14.38 [5.15]) than Drop‐Camera (mean = 1.00 [1.09]) or BRUV footage (mean = 8.42 [3.98]). Comparing the paired OceanBOSS Drop‐Camera and eDNA samples, 20 taxa were identified to the genera or species level from the Drop‐Camera, whereas 116 were identified with eDNA. The taxonomic resolution of the methods were similar, with 73.27% of fish taxa in eDNA resolved to the species level and 70% of taxa identified in Drop‐Camera footage. In the corresponding BRUV survey, 64 taxa were identified to genera or species level, with 82.8% being resolved to the species level (Table S2). An average abundance (site MaxN) of 3.29 [7.20] was observed using the Drop‐Camera method, whilst the BRUV method recorded an average of 28.7 [14.2].

**FIGURE 5:**
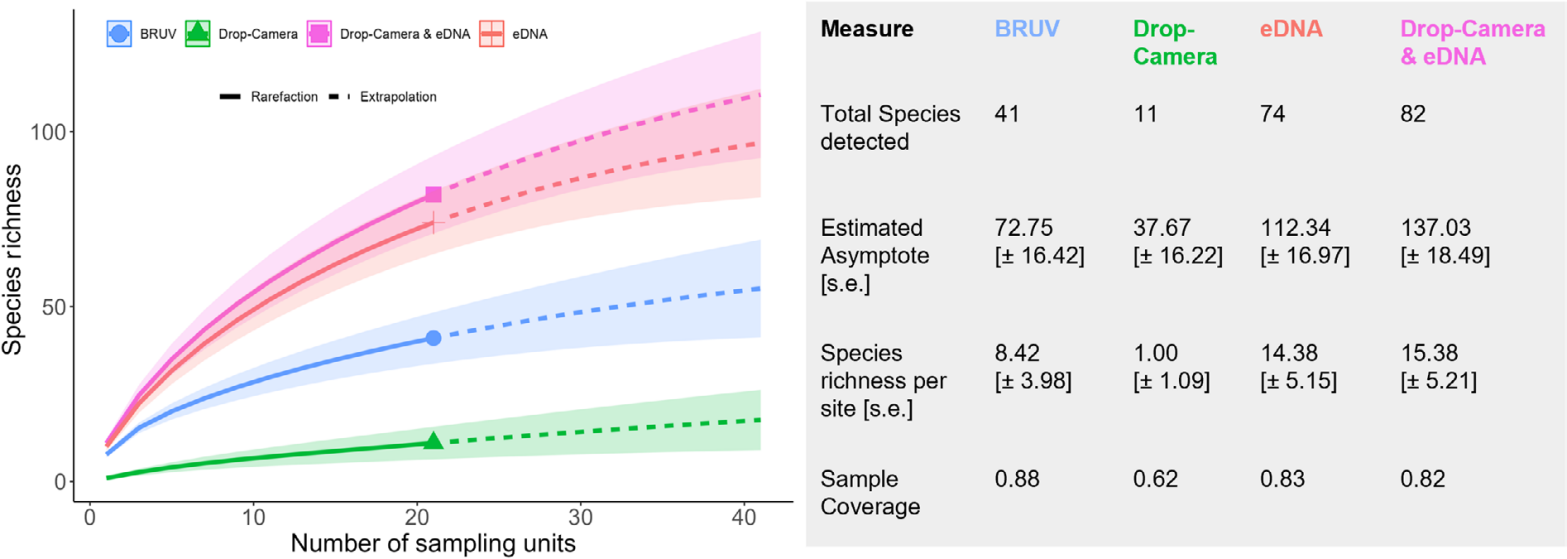
Left panel shows rarefaction (solid line) and extrapolation (dashed line) curves for each method, with the dashed lines indicating extrapolation of the sample size to 42. Right panel shows a table of results for each method, with total taxa detected including any detections to a genus or species level.

Species accumulation curves (Figure 5) showed the eDNA method to be closest to saturation (65.89% of asymptotic value), with Drop‐Camera and eDNA, BRUVs and Drop‐Camera species richness measurements being 59.85%, 56.48% and 29.18% of their estimated asymptotic values, respectively. Sample coverage was higher for BRUV (0.88) than eDNA (0.83) or Drop‐Camera (0.62). When combined, the paired OceanBOSS Drop‐Camera and eDNA observations increased species richness per site basis by 6.49% (Figure 5) over eDNA alone, along with an increase of 19.78% to the estimated asymptotic richness across all sites.

The composition of species was significantly different among methods (Table S4; Pseudo‐*F* (1–3) = 15.59, *p* < 0.001). All methods were significantly different from one another, with the largest average pairwise distance of 0.91 between BRUV and eDNA groups (*t* = 3.72, *p* < 0.001), whilst eDNA and Drop‐Camera had an average distance of 0.89 (*t* = 4.08, *p* < 0.001), and BRUV and Drop‐Camera groups were the closest at 0.67 (*t* = 4.07, *p* < 0.001) (Table S5). Mean within‐method distances were lowest for Drop‐Camera at 0.52, whilst BRUV and eDNA methods were higher with average distances of 0.67 and 0.76, respectively.

Concurrent CTD profiles were successfully acquired at all 21 sites using the OceanBOSS system (see Figure S5 for example). Across our sampling area, we observed various weak trends (Figures S2–S4), including a reduction in mean (*R* ^2^ = 0.272) and minimum (*R* ^2^ = 0.206) temperatures across our profiles with increasing depth. We also saw weak trends for increasing mean fluorescence with a higher latitude (*R* ^2^ = 0.171) and longitude (*R* ^2^ = 0.182), whilst the maximum recorded salinity decreased with an increasing latitude (*R* ^2^ = 0.195). Furthermore, we observed that during seabed landing the optical sensors responsible for measuring fluorescence/chlorophyll‐α and optical backscatter/turbidity exhibited minor but discernible interference (see Figure S5), which coincided with sediment disturbance observed in the cameras during contact with the seafloor.

## 4 Discussion

We have demonstrated a useful platform for the rapid collection of replicate water samples for eDNA extraction at depth that also can collect simultaneous stereo‐video imagery and biophysical oceanographic information. This OceanBOSS platform is amenable to spatially intensive sampling across a range of water depths and can be deployed in both the water column and to the seafloor. We successfully conducted testing of the OceanBOSS at Point Cloates, in the Ningaloo region of Western Australia, using equipment commonly found in oceanographic and marine imagery research facilities (i.e., CTD rosette and underwater cameras and/or camera housings), and analysed a total of 21 seafloor deployments, along with 21 nearby BRUV deployments for comparison.

In the development of eDNA methods, it is useful to collect paired or interspersed data from established methods in order to ground truth and start to understand biases in both the novel eDNA metabarcoding and established imagery datasets. Furthermore, its rectangular design also allows for safe and rapid deployment and retrieval using fish or crustacean‐trap retrieval equipment such as a davit arm or ‘pot tipper’ with hydraulic winch and is an ideal platform for working with local fisher‐skippers. The unbaited configuration tested has advantages that overcome complexities associated with using bait in close proximity to eDNA collection, including difficulties in determining the catchment area generated by bait‐plumes and the disproportionate attraction of high trophic‐level species (Dorman, Harvey, and Newman 2012; Hardinge et al. 2013), along with potential contamination of eDNA samples with high levels of DNA from the bait. In addition, the inclusion of multiple cameras to cover a wide field‐of‐view is known to aid the detection of shy or infrequently seen species (Whitmarsh, Huveneers, and Fairweather 2018).

We found the presence/absence assemblage sampled by each of the three methods to be significantly different from one another on a per site basis, with the eDNA metabarcoding and BRUV methods being most different, followed by eDNA metabarcoding and Drop‐Camera, whilst the Drop‐Camera and BRUV samples were the most similar. This corroborates previous research by Stat et al. (2019), which also demonstrated significant assemblage differences between eDNA metabarcoding and BRUV methods. However, both methods returned a comparable level of biodiversity, in contrast to our study, likely being due to analysis being conducted at the genus‐level and using a singular metabarcoding assay, in contrast to species‐level analyses and three assays in this study. Furthermore, a recent study in the United Kingdom (Clark et al. 2024) demonstrated that eDNA metabarcoding with two assays detected almost 3 times as many species as BRUVs, with BRUV data supplementing eDNA monitoring by recording species missed by eDNA and by providing additional environmental and life history metrics.

A significantly greater average abundance and richness of fish per site was observed with BRUVs than with the OceanBOSS' Drop‐Cameras, which may be a result of the longer sampling time of the BRUV methodology (1 h vs. 5 min), or increased fish recruitment to BRUVs due to the use of bait. However, the vast majority of species observed across all sites with the Drop‐Camera were also observed with the BRUVs which may suggest that the differences in richness are more likely attributed to the sampling duration rather than any inherent bias introduced by the use of the bait. The large discrepancy in the number of fish species detected between the Drop‐Camera and BRUV methods and the Drop‐Camera recording only eight species which were not detected with eDNA metabarcoding both highlight the comparative ineffectiveness of the unbaited Drop‐Camera method compared to the other methods used. Future research should therefore investigate longer deployment times and the use of attractants to aggregate fish in the field of view of the cameras in a manner similar to BRUVs.

Interestingly, the eDNA method detected a broad range of species that were not sampled by the imagery‐based methods, suggesting either that the effective sample unit size of the eDNA was much larger than that of the imagery methods, whereby the eDNA material detected may be travelling some distance to the sample location, or that juvenile life stages of these species, novel to the imagery data, were present in the local area but cryptic. For example, we noted fish species from the orders Gobiiformes (2), Myctophiformes (4) and Stomiiformes (1) which were only detected using eDNA. Underwater cameras also require the use of artificial light to work beyond a certain depth (such as those in this study) and at night, which can further affect the species' behaviour and thus detection rates. With eDNA metabarcoding, other studies that have surveyed fish have also shown that cryptic fish fauna can be readily identified using genetic methods (DiBattista et al. 2017; Stat et al. 2019). The detection of deep‐sea lanternfish (Myctophiformes) and dragonfish (Stomiiformes) illustrates the potential of eDNA metabarcoding to identify taxonomic groups that inhabit the mesopelagic and bathypelagic zone and are largely inaccessible with other monitoring techniques. These groups consist of small‐ to medium‐size predatory fish known to undertake diel vertical migration (Watanabe et al. 1999). Additionally, there are reports of Stomiiforme larvae developing in near‐surface waters prior to descent to meso‐ and bathypelagic depths (Fahay 2007); therefore, it is not unexpected that we detected these taxa in nearshore epipelagic waters. However, as noted in other studies (West et al. 2020; Hsu, Chen, and Denis 2023), this does raise the potential issue that, at present, there is not a way to discriminate between eDNA originating from eggs/larvae and mature fish.

We also note that 26.7% of fish taxa detected with eDNA metabarcoding could not be assigned to a species level, suggesting some insufficient resolution in our target metabarcoding regions for species‐level identification, or incomplete reference databases. PCR amplification biases due to primer selection as well as low copy number of eDNA templates can also lead to stochastic and preferential amplification of DNA from some species over others. For example, three species from the order Carchariniformes were identified from video recordings but not from eDNA, with each assay found to have a minimum of two mismatches with these species at each primer‐binding site, illustrating the challenges in detecting Chondrichthyes with assays designed for Actinopterygii (Miya et al. 2015; Stat et al. 2019).

Some studies indicate that 1 L water volume can adequately detect microorganisms of interest in some systems (Mächler et al. 2016); however, other studies suggest much larger volumes are required before species accumulation curves approach an asymptote for diversity (Koziol et al. 2018; Cantera et al. 2019; Hänfling et al. 2016). Furthermore, enhancing the sensitivity of an eDNA survey through increasing sampling volume may be desirable when the impact of reporting a false negative is high, such as in a biosecurity context. Filters may also become clogged with sediment or plankton/algae, reducing the volume that can be filtered, potentially resulting in large variability in the total filtered volume between replicates (Eichmiller, Bajer, and Sorensen 2014; Wittwer et al. 2019). We recommend that the ideal sampling volume for each site be determined with a pilot study, with future iterations of the OceanBOSS potentially requiring a larger frame size to accomodate larger volume or a greater number of Niskin bottles.

This study provides researchers with a novel tool for concurrently collecting stereo‐video imagery and water samples and may assist future researchers with challenges associated with the application of eDNA data for environmental reporting. The first challenge involves understanding the spatial extent represented by an eDNA sample, particularly in the marine environment where genetic materials can be transported over considerable distances to the sampling location (Andruszkiewicz et al. 2019). The second challenge pertains to quantifying the relative abundance of an organism by age or body size class, which the imagery methods provided but was not examined in this research. The third challenge relates to the time‐consuming process of filtering seawater; although our system demonstrated the capability to complete up to 40 deployments per day within our designated test area (Figure 3), eDNA sample collection rates were constrained by the time required to filter each set of seawater samples. Future research should look to improve the throughput of filtration methods or advance filtration‐independent sampling methods whilst benchmarking their efficacy against filtration‐based methods.

## Author Contributions

**Samuel Thompson:** conceptualization (lead), data curation (lead), formal analysis (lead), investigation (lead), methodology (lead), software (lead), writing – original draft (lead). **Simon Jarman:** funding acquisition (equal), project administration (equal), supervision (equal), va writing – review and editing (equal). **Kingsley Griffin:** data curation (equal), formal analysis (supporting), supervision (equal), writing – review and editing (equal). **Claude Spencer:** data curation (supporting), writing – review and editing (supporting). **Gabrielle Cummins:** data curation (supporting), methodology (supporting), writing – review and editing (supporting). **Julian Partridge:** funding acquisition (equal), supervision (equal), validation (equal), writing – review and editing (equal). **Tim Langlois:** funding acquisition (equal), supervision (lead), writing – original draft (equal), writing – review and editing (lead).

## Conflicts of Interest

The authors declare no conflicts of interest.

## Supporting information


Data S1.

## Data Availability

Raw sequence reads are deposited in the NCBI sequence read archive (BioProject PRJNA1106763). Raw EventMeasure outputs are deposited in GlobalArchive (Langlois and Friedman 2019), under the Campaign ID: ‘2022‐05_PtCloates_stereo‐BRUVS’ for the Stereo‐BRUV dataset and ‘2022‐05_PtCloates_stereo‐BOSS’ for the Drop‐Camera dataset.
