# Supplementary material for: Novel Drop‐Sampler for Simultaneous Collection of Stereo‐Video, Environmental DNA and Oceanographic Data
Source: Ecol Evol. 2024 Dec 23;14(12):e70705. doi: 10.1002/ece3.70705 (PMC11664325; doi:10.1002/ece3.70705)
Supplement: Supplementary file 1 — Data S1. [file ECE3-14-e70705-s001.docx]

A novel drop-sampler for simultaneous collection of stereo-video, environmental DNA and oceanographic data: Supplementary information

## **Figure S1:** PCR protocols

**PCR Indexing strategy**

Indexes = 10 bp (could be less or more)

P5 Index Forward sequencing pb site Round1 forward primer sequence

5’ AATGATACGGCGACCACCGAGATCTACACXXXXXXXXXXTCGTCGGCAGCGTC 3’

5’ TCGTCGGCAGCGTCAGATGTGTATAAGAGACAGXXXXXXXXXXXXXXXXXXXX 3’

P7 Index Reverse sequencing pb site Round1 reverse primer sequence

5’ CAAGCAGAAGACGGCATACG*A*GATXXXXXXXXXXGTCTCGTGGGCTCGG 3’

5’ GTCTCGTGGGCTCGGAGATGTGTATAAGAGACAGXXXXXXXXXXXXXXXXXXXX 3’

**1^st^ round thermal cycling - 10 μL reaction volume**

5μL PowerUp SYBR green master mix (2x), 0.25μL of each 10uM primer, 0.1μL BSA, 4.4μL DNA extraction

50°C 5 min

95°C 10 min

**16S-Lutjanidae 16S-Fish 16S-** **Fish_SynShort**

(95°C 30 s, 65°C 1 min) x50 (95°C 30 s, 54°C 30 sec, 72°C 45 sec) x50 (95°C 30 s, 55°C 30 sec, 72°C 45 sec) x50

72°C 10 min 72°C 10 min 72°C 10 min

**2^nd^ round thermal cycling - 10 μL reaction volume**

5μL PowerUp SYBR green master mix (2x), 1uL of each primer at 10μM, 1μL 1^st^ round amplicons, 2μl H_2_O

50°C 5 min

95°C 10 min

(95°C 30 s, 65°C 1 min) x10

65°C 10 min

##
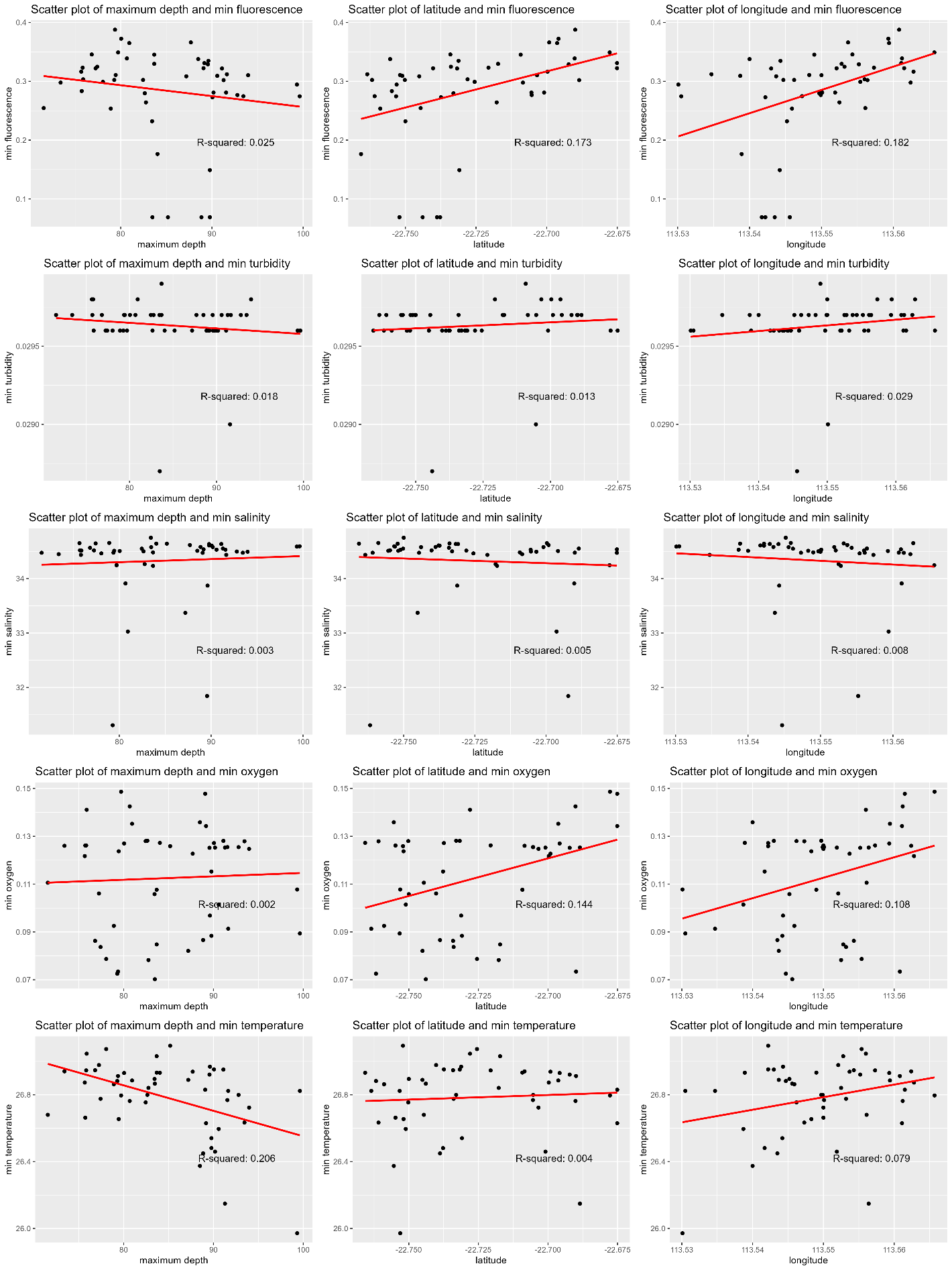
**Figure S2:** Minimum CTD values for temperature (ITS-90, °C) and salinity (practical salinity units), fluorescence (chlorophyll-α µg/µL), turbidity (FTU) and dissolved oxygen. Temperature and salinity data was collected using an SBE19plus CTD (Sea-Bird Scientific, Washington, USA), additional sensors include an in-situ fluorometer Chelsea MiniTracka II (Chelsea technologies, Surrey, UK), a Chelsea MiniTracka II nephelometer (Chelsea technologies, Surrey, UK), and an SBE43 dissolved oxygen (DO) sensor (Sea-Bird Scientific, Washington, USA).

##
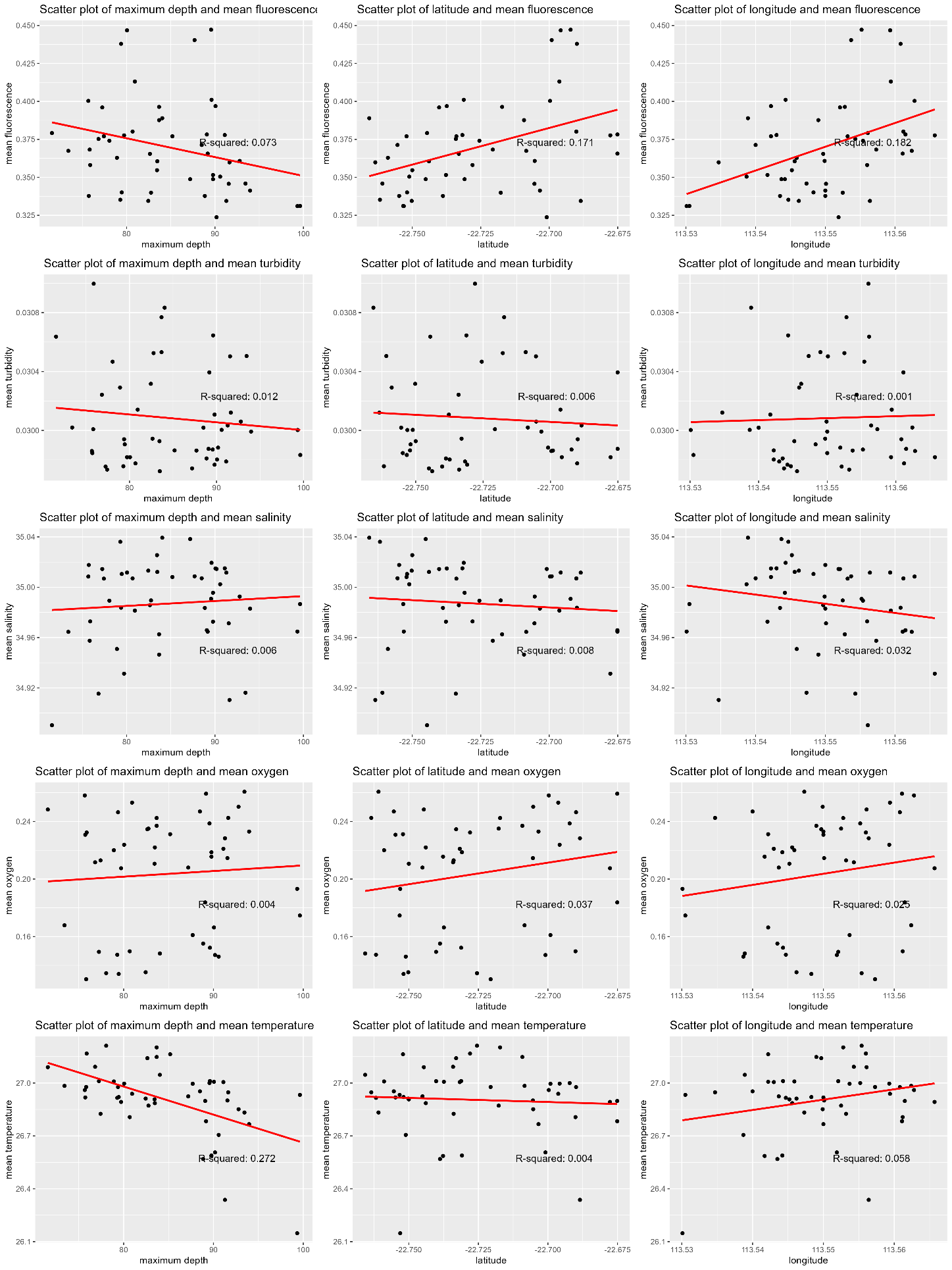
**Figure S3:** Mean CTD values for temperature (ITS-90, °C) and salinity (practical salinity units), fluorescence (chlorophyll-α µg/µL), turbidity (FTU) and dissolved oxygen. Temperature and salinity data was collected using an SBE19plus CTD (Sea-Bird Scientific, Washington, USA), additional sensors include an in-situ fluorometer Chelsea MiniTracka II (Chelsea technologies, Surrey, UK), a Chelsea MiniTracka II nephelometer (Chelsea technologies, Surrey, UK), and an SBE43 dissolved oxygen (DO) sensor (Sea-Bird Scientific, Washington, USA).

##
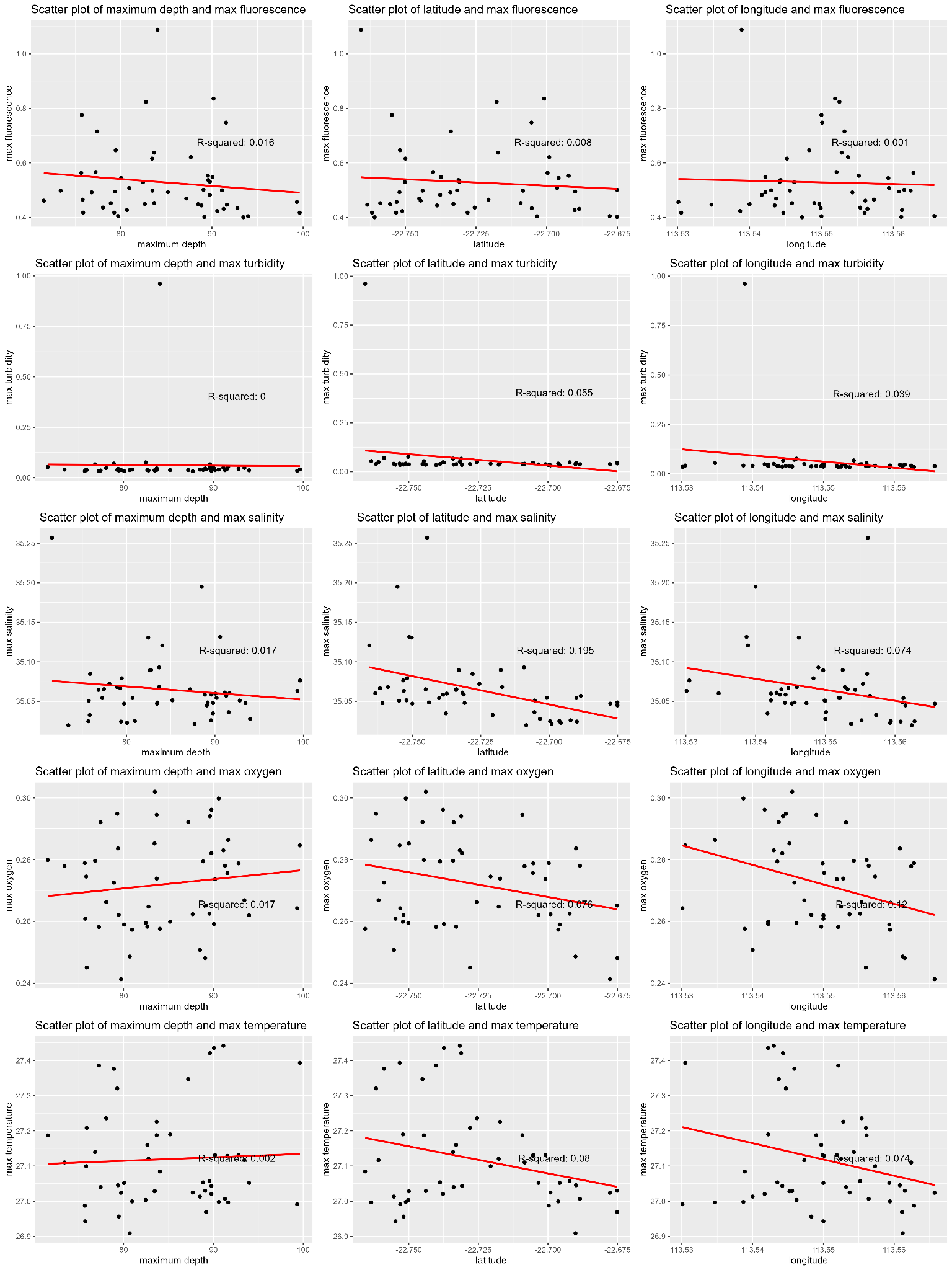
**Figure S4:** Maximum CTD values for temperature (ITS-90, °C) and salinity (practical salinity units), fluorescence (chlorophyll-α µg/µL), turbidity (FTU) and dissolved oxygen. Temperature and salinity data was collected using an SBE19plus CTD (Sea-Bird Scientific, Washington, USA), additional sensors include an in-situ fluorometer Chelsea MiniTracka II (Chelsea technologies, Surrey, UK), a Chelsea MiniTracka II nephelometer (Chelsea technologies, Surrey, UK), and an SBE43 dissolved oxygen (DO) sensor (Sea-Bird Scientific, Washington, USA).

##
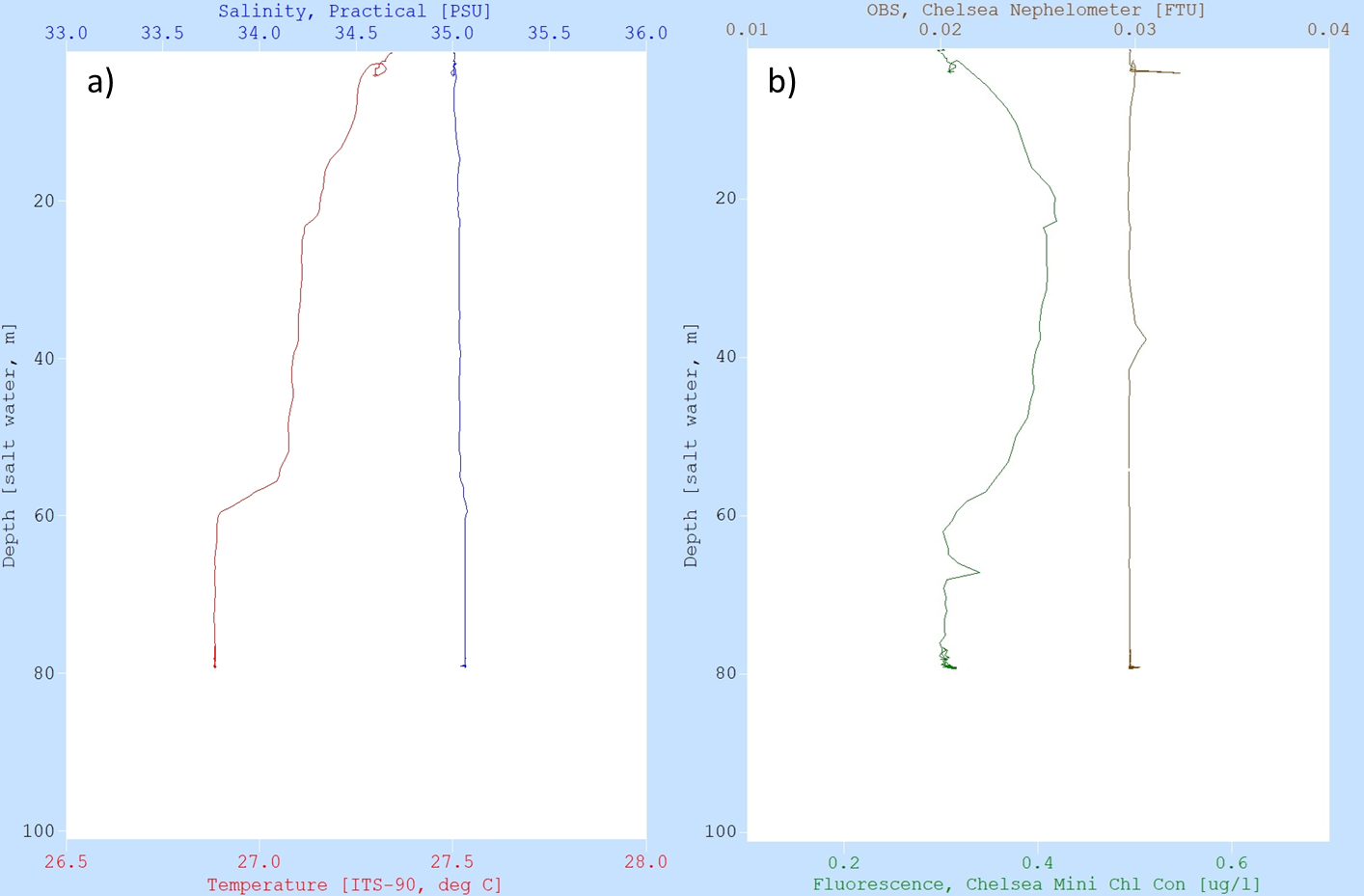
Figure S5: An example CTD profile (downcast only) collected at Point Cloates. Profiles were collected using a Sea-Bird Scientific SBE 19plusV2 CTD with additional in-situ Chelsea MiniTracka II fluorometer and Chelsea MiniTracka II nephelometer for measuring optical backscatter as a proxy for water turbidity. Panel a) shows example temperature and salinity profiles, whilst panel b) shows example chlorophyll-α and optical backscatter sensor (OBS) profiles from the same site.

## Appendix S1: 16S-Lutjanidae assay

Several PCR metabarcoding assays were designed to target the 16S rRNA region of the mitochondrial genome of Lutjanidae species. Publicly available (NCBI) reference sequences for Lutjanidae species were aligned in AliView v 1.27 ([Larsson 2014](https://paperpile.com/c/iCoGXC/Kcra)) to identify short, conserved regions capable of amplifying degraded DNA fragments commonly found in environmental samples.

Each assay was designed based on the following criteria: 18 - 24 bp in length, balanced GC content (40 - 60 %), free from secondary structures and similar annealing temperatures on forward and reverse primers. To determine the efficacy, each assay was tested in-vitro using quantitative PCR (qPCR) of tissue and environmental samples. Reactions were performed using neat and a two point tenfold dilution of tissue samples (species: *L. sebae*, *L. malabaricus*, *L. erythropterus*, *L russelli, L argentimaculatus, P. multidens*) obtained from frames donated from local fish markets (Perth Seafoods and Sealanes). One optimal assay set (termed 16S-Lutjanidae) was selected for further testing based on its reliability to amplify Lutjanidae mtDNA in tissue samples. This assay was used for the study along with 16S-Fish and 16S-FishSynShort assays.

## **Table S1 |** Sampling details of the metabarcoding study including unique Sample ID’s, depth (m), latitude and longitude (decimal degrees), date and time, fishing status of the area, and sampling method. All sampling was conducted over a week period in May 2022 onboard the vessel Keshi Mer, at Point Cloates, Western Australia.

| **Sample ID** | **Depth (m)** | **Latitude** | **Longitude** | **Date + Time** | **Fishing status** | **Sampling Method** |
| --- | --- | --- | --- | --- | --- | --- |
| 120 | 75.2 | -22.6997 | 113.5628 | 26/05/2023 T10:36:07+08:00 | No-take | Drop-Camera + eDNA |
| 125 | 84.7 | -22.7521 | 113.5422 | 23/05/2023 T11:51:02+08:00 | No-take | Drop-Camera + eDNA |
| 133 | 87.5 | -22.7256 | 113.5479 | 23/05/2023 T15:00:34+08:00 | No-take | Drop-Camera + eDNA |
| 139 | 92.2 | -22.7092 | 113.549 | 25/05/2023 T8:16:36+08:00 | No-take | Drop-Camera + eDNA |
| 140 | 88.6 | -22.6991 | 113.5537 | 26/05/2023 T12:03:42+08:00 | No-take | Drop-Camera + eDNA |
| 149 | 94.5 | -22.7034 | 113.55 | 25/05/2023 T11:24:56+08:00 | No-take | Drop-Camera + eDNA |
| 152 | 77.1 | -22.7255 | 113.5554 | 23/05/2023 T13:41:00+08:00 | No-take | Drop-Camera + eDNA |
| 212 | 75 | -22.728 | 113.556 | 23/05/2023 T11:00:30+08:00 | No-take | Drop-Camera + eDNA |
| 214 | 81.5 | -22.733 | 113.5497 | 23/05/2023 T9:52:30+08:00 | No-take | Drop-Camera + eDNA |
| 225 | 72.1 | -22.7084 | 113.5624 | 25/05/2023 T14:33:25+08:00 | No-take | Drop-Camera + eDNA |
| 230 | 76.9 | -22.7341 | 113.5543 | 23/05/2023 T8:31:26+08:00 | No-take | Drop-Camera + eDNA |
| 239 | 90.5 | -22.6885 | 113.5564 | 26/05/2023 T16:04:43+08:00 | No-take | Drop-Camera + eDNA |
| 243 | 74.4 | -22.7206 | 113.5573 | 25/05/2023 T13:14:20+08:00 | No-take | Drop-Camera + eDNA |
| 248 | 76.6 | -22.7609 | 113.5473 | 19/05/2023 T8:27:25+08:00 | No-take | Drop-Camera + eDNA |
| 259 | 88.9 | -22.7312 | 113.5443 | 21/05/2023 T16:24:58+08:00 | No-take | Drop-Camera + eDNA |
| 260 | 83.6 | -22.7177 | 113.5525 | 25/05/2023 T13:53:04+08:00 | No-take | Drop-Camera + eDNA |
| 261 | 78.8 | -22.6899 | 113.5608 | 26/05/2023 T15:21:00+08:00 | No-take | Drop-Camera + eDNA |
| B48 | 79.6 | -22.6963 | 113.5594 | 26/05/2023 T11:21:54+08:00 | No-take | Drop-Camera + eDNA |
| B54 | 84 | -22.7654 | 113.5391 | 21/05/2023 T11:38:15+08:00 | No-take | Drop-Camera + eDNA |
| B58 | 92.5 | -22.7053 | 113.5499 | 25/05/2023 T9:56:58+08:00 | No-take | Drop-Camera + eDNA |
| B61 | 78.8 | -22.6777 | 113.5657 | 27/05/2023 T11:52:52+08:00 | No-take | Drop-Camera + eDNA |
| 4 | 73.9 | -22.7337 | 113.5555 | 20/05/2023 T14:40:43+08:00 | No-take | BRUV |
| 11 | 81 | -22.7274 | 113.5498 | 22/05/2023 T08:18:17+08:00 | No-take | BRUV |
| 14 | 90.5 | -22.6876 | 113.5568 | 26/05/2023 T08:48:10+08:00 | No-take | BRUV |
| 17 | 86.5 | -22.7011 | 113.5532 | 22/05/2023 T12:45:32+08:00 | No-take | BRUV |
| 18 | 91.5 | -22.7101 | 113.5492 | 22/05/2023 T10:29:53+08:00 | No-take | BRUV |
| 20 | 76 | -22.7212 | 113.5561 | 22/05/2023 T08:44:53+08:00 | No-take | BRUV |
| 26 | 77.2 | -22.7615 | 113.5484 | 20/05/2023 T09:08:31+08:00 | No-take | BRUV |
| 27 | 90.4 | -22.7254 | 113.5459 | 22/05/2023 T08:31:16+08:00 | No-take | BRUV |
| 28 | 76.3 | -22.6987 | 113.5625 | 22/05/2023 T13:05:17+08:00 | No-take | BRUV |
| 29 | 87.9 | -22.691 | 113.5564 | 22/05/2023 T14:54:59+08:00 | No-take | BRUV |
| 31 | 82.3 | -22.7542 | 113.5424 | 20/05/2023 T10:27:48+08:00 | No-take | BRUV |
| 32 | 78.3 | -22.7239 | 113.5552 | 22/05/2023 T08:39:35+08:00 | No-take | BRUV |
| 36 | 83.2 | -22.7318 | 113.5477 | 20/05/2023 T14:25:23+08:00 | No-take | BRUV |
| 40 | 84.1 | -22.7522 | 113.5444 | 20/05/2023 T11:58:07+08:00 | No-take | BRUV |
| 48 | 80.2 | -22.696 | 113.5596 | 22/05/2023 T14:21:46+08:00 | No-take | BRUV |
| 50 | 81.9 | -22.7173 | 113.5528 | 22/05/2023 T10:14:30+08:00 | No-take | BRUV |
| 51 | 79.4 | -22.69 | 113.5609 | 22/05/2023 T14:49:13+08:00 | No-take | BRUV |
| 54 | 83.1 | -22.7654 | 113.5392 | 20/05/2023 T08:32:36+08:00 | No-take | BRUV |
| 58 | 90.9 | -22.7056 | 113.5502 | 22/05/2023 T12:28:44+08:00 | No-take | BRUV |
| 61 | 79.8 | -22.6779 | 113.5654 | 26/05/2023 T08:23:34+08:00 | No-take | BRUV |
| 64 | 79.2 | -22.7343 | 113.5508 | 20/05/2023 T14:30:16+08:00 | No-take | BRUV |

##

## **Table S2 |** List of all *Actinopterygii* and *Chondrichthyes* species included in the analysis, and which method(s) yielded a detection.

| **Class** | **Order** | **Family** | **Genus** | **Species** | **eDNA** | **BRUV** | **Drop-**  **Camera** |
| --- | --- | --- | --- | --- | --- | --- | --- |
| Chondrichthyes | Carcharhiniformes | Carcharhinidae | Carcharhinus | plumbeus |  |  | X |
| Actinopterygii | Acanthuriformes | Acanthuridae | Naso | brachycentron |  | X |  |
| Actinopterygii | Acanthuriformes | Acanthuridae | Naso | fageni |  | X |  |
| Actinopterygii | Acanthuriformes | Acanthuridae | Naso | hexacanthus |  | X |  |
| Actinopterygii | Carangiformes | Carangidae | Carangoides | chrysophrys |  | X |  |
| Actinopterygii | Carangiformes | Carangidae | Carangoides | gymnostethus |  | X |  |
| Actinopterygii | Carangiformes | Carangidae | Caranx | lugubris |  | X |  |
| Actinopterygii | Carangiformes | Carangidae | Seriola | dumerili |  | X |  |
| Actinopterygii | Chaetodontiformes | Chaetodontidae | Chaetodon | assarius |  | X |  |
| Actinopterygii | Lutjaniformes | Lutjanidae | Lutjanus | lemniscatus |  | X |  |
| Actinopterygii | Perciformes | Mullidae | Parupeneus | cyclostomus |  | X |  |
| Actinopterygii | Perciformes | Mullidae | Parupeneus | heptacanthus |  | X |  |
| Actinopterygii | Perciformes | Mullidae | Upeneus | moluccensis |  | X |  |
| Actinopterygii | Perciformes | Scaridae | Scarus | ghobban |  | X |  |
| Actinopterygii | Perciformes | Serranidae | Epinephelus | areolatus |  | X |  |
| Actinopterygii | Perciformes | Serranidae | Epinephelus | coioides |  | X |  |
| Actinopterygii | Perciformes | Serranidae | Epinephelus | multinotatus |  | X |  |
| Actinopterygii | Perciformes | Sphyraenidae | Sphyraena | jello |  | X |  |
| Actinopterygii | Scombriformes | Scombridae | Scomberomorus | commerson |  | X |  |
| Actinopterygii | Tetraodontiformes | Balistidae | Pseudobalistes | flavimarginatus |  | X |  |
| Actinopterygii | Tetraodontiformes | Tetraodontidae | Arothron | stellatus |  | X |  |
| Actinopterygii | Tetraodontiformes | Tetraodontidae | Lagocephalus | sceleratus |  | X |  |
| Chondrichthyes | Carcharhiniformes | Sphyrnidae | Sphyrna | mokarran |  | X |  |
| Actinopterygii | Acanthuriformes | Acanthuridae | Acanthurus | mata | X |  |  |
| Actinopterygii | Acanthuriformes | Acanthuridae | Acanthurus | triostegus | X |  |  |
| Actinopterygii | Acanthuriformes | Acanthuridae | Naso | mcdadei | X |  |  |
| Actinopterygii | Acanthuriformes | Acanthuridae | Naso | unicornis | X |  |  |
| Actinopterygii | Anguilliformes | Muraenidae | Gymnothorax | eurostus | X |  |  |
| Actinopterygii | Anguilliformes | Muraenidae | Gymnothorax | fuscomaculatus | X |  |  |
| Actinopterygii | Anguilliformes | Muraenidae | Gymnothorax | mucifer | X |  |  |
| Actinopterygii | Anguilliformes | Muraenidae | Gymnothorax | thyrsoideus | X |  |  |
| Actinopterygii | Aulopiformes | Synodontidae | Saurida | nebulosa | X |  |  |
| Actinopterygii | Aulopiformes | Synodontidae | Synodus | kaianus | X |  |  |
| Actinopterygii | Aulopiformes | Synodontidae | Trachinocephalus | myops | X |  |  |
| Actinopterygii | Carangiformes | Carangidae | Atule | mate | X |  |  |
| Actinopterygii | Carangiformes | Carangidae | Caranx | ignobilis | X |  |  |
| Actinopterygii | Carangiformes | Carangidae | Caranx | melampygus | X |  |  |
| Actinopterygii | Carangiformes | Carangidae | Decapterus | macarellus | X |  |  |
| Actinopterygii | Carangiformes | Carangidae | Elagatis | bipinnulata | X |  |  |
| Actinopterygii | Carangiformes | Carangidae | Selar | crumenophthalmus | X |  |  |
| Actinopterygii | Carangiformes | Carangidae | Seriola | lalandi | X |  |  |
| Actinopterygii | Centrarchiformes | Kyphosidae | Kyphosus | bigibbus | X |  |  |
| Actinopterygii | Centrarchiformes | Percichthyidae | Percalates | novemaculeata | X |  |  |
| Actinopterygii | Chaetodontiformes | Chaetodontidae | Chaetodon | kleinii | X |  |  |
| Actinopterygii | Gobiiformes | Gobiidae | Gobiodon | axillaris | X |  |  |
| Actinopterygii | Gobiiformes | Gobiidae | Valenciennea | helsdingenii | X |  |  |
| Actinopterygii | Holocentriformes | Holocentridae | Myripristis | kuntee | X |  |  |
| Actinopterygii | Holocentriformes | Holocentridae | Sargocentron | rubrum | X |  |  |
| Actinopterygii | Kurtiformes | Apogonidae | Apogon | semilineatus | X |  |  |
| Actinopterygii | Labriformes | Labridae | Choerodon | cauteroma | X |  |  |
| Actinopterygii | Labriformes | Labridae | Choerodon | rubescens | X |  |  |
| Actinopterygii | Labriformes | Labridae | Cymolutes | praetextatus | X |  |  |
| Actinopterygii | Labriformes | Labridae | Halichoeres | nebulosus | X |  |  |
| Actinopterygii | Labriformes | Labridae | Iniistius | pavo | X |  |  |
| Actinopterygii | Labriformes | Labridae | Oxycheilinus | bimaculatus | X |  |  |
| Actinopterygii | Labriformes | Labridae | Oxycheilinus | orientalis | X |  |  |
| Actinopterygii | Labriformes | Labridae | Scarus | niger | X |  |  |
| Actinopterygii | Labriformes | Labridae | Scarus | schlegeli | X |  |  |
| Actinopterygii | Lutjaniformes | Haemulidae | Plectorhinchus | flavomaculatus | X |  |  |
| Actinopterygii | Lutjaniformes | Haemulidae | Plectorhinchus | pictus | X |  |  |
| Actinopterygii | Lutjaniformes | Lutjanidae | Lutjanus | monostigma | X |  |  |
| Actinopterygii | Lutjaniformes | Lutjanidae | Pristipomoides | filamentosus | X |  |  |
| Actinopterygii | Lutjaniformes | Lutjanidae | Pristipomoides | multidens | X |  |  |
| Actinopterygii | Lutjaniformes | Lutjanidae | Pterocaesio | digramma | X |  |  |
| Actinopterygii | Myctophiformes | Myctophidae | Benthosema | fibulatum | X |  |  |
| Actinopterygii | Myctophiformes | Myctophidae | Benthosema | pterotum | X |  |  |
| Actinopterygii | Myctophiformes | Myctophidae | Dasyscopelus | selenops | X |  |  |
| Actinopterygii | Myctophiformes | Myctophidae | Diaphus | garmani | X |  |  |
| Actinopterygii | Perciformes | Mullidae | Mulloidichthys | vanicolensis | X |  |  |
| Actinopterygii | Perciformes | Mullidae | Parupeneus | pleurostigma | X |  |  |
| Actinopterygii | Perciformes | Mullidae | Upeneus | tragula | X |  |  |
| Actinopterygii | Perciformes | Platycephalidae | Sorsogona | tuberculata | X |  |  |
| Actinopterygii | Perciformes | Pomacanthidae | Centropyge | tibicen | X |  |  |
| Actinopterygii | Perciformes | Pomacentridae | Plectroglyphidodon | dickii | X |  |  |
| Actinopterygii | Perciformes | Pomacentridae | Stegastes | nigricans | X |  |  |
| Actinopterygii | Perciformes | Serranidae | Cephalopholis | sexmaculata | X |  |  |
| Actinopterygii | Perciformes | Serranidae | Cephalopholis | sonnerati | X |  |  |
| Actinopterygii | Perciformes | Serranidae | Epinephelus | bleekeri | X |  |  |
| Actinopterygii | Spariformes | Lethrinidae | Gymnocranius | microdon | X |  |  |
| Actinopterygii | Spariformes | Lethrinidae | Lethrinus | olivaceus | X |  |  |
| Actinopterygii | Stomiiformes | Phosichthyidae | Vinciguerria | nimbaria | X |  |  |
| Actinopterygii | Tetraodontiformes | Monacanthidae | Paramonacanthus | choirocephalus | X |  |  |
| Actinopterygii | Tetraodontiformes | Monacanthidae | Pervagor | janthinosoma | X |  |  |
| Actinopterygii | Tetraodontiformes | Ostraciidae | Lactoria | fornasini | X |  |  |
| Chondrichthyes | Myliobatiformes | Dasyatidae | Neotrygon | kuhlii | X |  |  |
| Actinopterygii | Carangiformes | Carangidae | Carangoides | fulvoguttatus |  | X | X |
| Actinopterygii | Labriformes | Labridae | Coris | caudimacula |  | X | X |
| Actinopterygii | Lutjaniformes | Lutjanidae | Lutjanus | sebae | X | X | X |
| Actinopterygii | Perciformes | Cirrhitidae | Paracirrhites | forsteri |  | X | X |
| Actinopterygii | Perciformes | Malacanthidae | Malacanthus | brevirostris |  | X | X |
| Actinopterygii | Perciformes | Pinguipedidae | Parapercis | nebulosa |  | X | X |
| Actinopterygii | Spariformes | Lethrinidae | Lethrinus | miniatus | X | X | X |
| Actinopterygii | Spariformes | Lethrinidae | Lethrinus | rubrioperculatus |  | X | X |
| Actinopterygii | Spariformes | Nemipteridae | Pentapodus | nagasakiensis | X | X | X |
| Chondrichthyes | Carcharhiniformes | Carcharhinidae | Carcharhinus | albimarginatus |  | X | X |
| Actinopterygii | Anguilliformes | Muraenidae | Gymnothorax | pseudothyrsoideus | X | X |  |
| Actinopterygii | Chaetodontiformes | Chaetodontidae | Heniochus | acuminatus | X | X |  |
| Actinopterygii | Lutjaniformes | Lutjanidae | Aphareus | rutilans | X | X |  |
| Actinopterygii | Perciformes | Pomacanthidae | Pomacanthus | semicirculatus | X | X |  |
| Actinopterygii | Perciformes | Serranidae | Variola | louti | X | X |  |
| Actinopterygii | Spariformes | Lethrinidae | Gymnocranius | grandoculis | X | X |  |
| Actinopterygii | Spariformes | Lethrinidae | Lethrinus | nebulosus | X | X |  |
| Actinopterygii | Syngnathiformes | Fistulariidae | Fistularia | commersonii | X | X |  |
| Actinopterygii | Tetraodontiformes | Balistidae | Sufflamen | fraenatum | X | X |  |

## **Table S3 |** List of all *Actinopterygii* and *Chondrichthyes* taxa removed from the analysis due to being unresolved to a species level ( *^1^* ), due to having no occurrence records for the area ( *^2^* ) in the Atlas of Living Australia ([Belbin et al. 2021](https://paperpile.com/c/iCoGXC/bLNT)), not having a reference genome available ( *^3^* ) or not having a reference genome available for other members of the genera ( *^4^* ).

| **Class** | **Order** | **Family** | **Genus** | **Species** | **eDNA** | **BRUV** | **Drop- Camera** |
| --- | --- | --- | --- | --- | --- | --- | --- |
| Actinopterygii | Perciformes | Pomacentridae | Amblypomacentrus | breviceps^4^ |  |  | X |
| Actinopterygii | Labriformes | Labridae | Xenojulis | margaritaceus^4^ | X |  |  |
| Actinopterygii | Lutjaniformes | Lutjanidae | Symphorus | nematophorus^4^ | X |  |  |
| Actinopterygii | Carangiformes | Carangidae | Seriolina | nigrofasciata^4^ |  | X |  |
| Actinopterygii | Perciformes | Pomacentridae | Pristotis | obtusirostris^4^ | X |  |  |
| Actinopterygii | Perciformes | Sparidae | Argyrops | spinifer^4^ |  | X |  |
| Actinopterygii | Lutjaniformes | Lutjanidae | Aprion | virescens^4^ | X |  |  |
| Actinopterygii | Lutjaniformes | Lutjanidae | Paracaesio | xanthura^4^ | X |  |  |
| Actinopterygii | Perciformes | Cirrhitidae | Cirrhitichthys | aprinus^4^ |  | X |  |
| Actinopterygii | Beloniformes | Exocoetidae | Parexocoetus | brachypterus^4^ | X |  |  |
| Actinopterygii | Carangiformes | Carangidae | Megalaspis | cordyla^4^ | X |  |  |
| Actinopterygii | Tetraodontiformes | Balistidae | Pseudobalistes | fuscus^4^ | X |  |  |
| Actinopterygii | Labriformes | Labridae | Cheilio | inermis^4^ | X |  |  |
| Chondrichthyes | Carcharhiniformes | Carcharhinidae | Loxodon | macrorhinus^4^ |  | X |  |
| Actinopterygii | Labriformes | Labridae | Bolbometopon | muricatum^4^ | X |  |  |
| Actinopterygii | Perciformes | Echeneidae | Echeneis | naucrates^4^ |  | X | X |
| Actinopterygii | Lutjaniformes | Haemulidae | Diagramma | pictum labiosum^4^ |  | X |  |
| Chondrichthyes | Orectolobiformes | Stegostomatidae | Stegostoma | tigrinum^4^ |  | X |  |
| Actinopterygii | Labriformes | Labridae | Leptoscarus | vaigiensis^4^ | X |  |  |
| Actinopterygii | Labriformes | Labridae | Suezichthys | cyanolaemus^3^ |  | X |  |
| Actinopterygii | Spariformes | Lethrinidae | Gymnocranius | euanus^3^ |  | X |  |
| Actinopterygii | Tetraodontiformes | Balistidae | Abalistes | filamentosus^3^ |  | X |  |
| Actinopterygii | Tetraodontiformes | Balistidae | Xanthichthys | lineopunctatus^3^ |  |  | X |
| Actinopterygii | Spariformes | Lethrinidae | Lethrinus | ravus^3^ |  | X |  |
| Actinopterygii | Labriformes | Labridae | Bodianus | solatus^3^ |  | X |  |
| Actinopterygii | Carangiformes | Carangidae | Carangoides | dropped^1^ |  | X |  |
| Actinopterygii | Carangiformes | Carangidae | Decapterus | dropped^1^ |  | X |  |
| Actinopterygii | Labriformes | Labridae | Choerodon | dropped^1^ |  | X |  |
| Actinopterygii | Perciformes | Scaridae | Scarus | dropped^1^ |  | X |  |
| Actinopterygii | Scombriformes | Scombridae | Scomberomorus | dropped^1^ |  | X |  |
| Actinopterygii | Acanthuriformes | Acanthuridae | Acanthurus | dropped^1^ | X |  |  |
| Actinopterygii | Acanthuriformes | Acanthuridae | Ctenochaetus | dropped^1^ | X |  |  |
| Actinopterygii | Acanthuriformes | Acanthuridae | Naso | dropped^1^ | X |  |  |
| Actinopterygii | Carangiformes | Carangidae | Caranx | dropped^1^ | X |  |  |
| Actinopterygii | Carangiformes | Carangidae | Gnathanodon | dropped^1^ | X |  |  |
| Actinopterygii | Carangiformes | Carangidae | Seriola | dropped^1^ | X |  |  |
| Actinopterygii | Centrarchiformes | Kyphosidae | Kyphosus | dropped^1^ | X |  |  |
| Actinopterygii | Gobiiformes | Gobiidae | Amblygobius | dropped^1^ | X |  |  |
| Actinopterygii | Gobiiformes | Gobiidae | Ptereleotris | dropped^1^ | X |  |  |
| Actinopterygii | Holocentriformes | Holocentridae | Myripristis | dropped^1^ | X |  |  |
| Actinopterygii | Istiophoriformes | Sphyraenidae | Sphyraena | dropped^1^ | X |  |  |
| Actinopterygii | Kurtiformes | Apogonidae |  | dropped^1^ | X |  |  |
| Actinopterygii | Labriformes | Labridae | Cheilinus | dropped^1^ | X |  |  |
| Actinopterygii | Labriformes | Labridae | Scarus | dropped^1^ | X |  |  |
| Actinopterygii | Labriformes | Labridae | Stethojulis | dropped^1^ | X |  |  |
| Actinopterygii | Lutjaniformes | Lutjanidae | Lutjanus | dropped^1^ | X |  |  |
| Actinopterygii | Lutjaniformes | Lutjanidae | Pterocaesio | dropped^1^ | X |  |  |
| Actinopterygii | Myctophiformes | Myctophidae | Diaphus | dropped^1^ | X |  |  |
| Actinopterygii | Perciformes | Pomacentridae | Pomacentrus | dropped^1^ | X |  |  |
| Actinopterygii | Perciformes | Siganidae | Siganus | dropped^1^ | X |  |  |
| Actinopterygii | Scombriformes | Nomeidae | Cubiceps | dropped^1^ | X |  |  |
| Actinopterygii | Scombriformes | Scombridae | Auxis | dropped^1^ | X |  |  |
| Actinopterygii | Scombriformes | Trichiuridae | Trichiurus | dropped^1^ | X |  |  |
| Actinopterygii | Siluriformes | Plotosidae | Plotosus | dropped^1^ | X |  |  |
| Actinopterygii | Spariformes | Sparidae | Argyrops | dropped^1^ | X |  |  |
| Actinopterygii | Spariformes | Sparidae | Pagrus | dropped^1^ | X |  |  |
| Chondrichthyes | Myliobatiformes | Dasyatidae | Urogymnus | dropped^1^ | X |  |  |
| Chondrichthyes | Myliobatiformes | Myliobatidae | Mobula | dropped^1^ | X |  |  |
| Actinopterygii | Lutjaniformes | Lutjanidae | Pristipomoides | dropped^1^ |  | X | X |
| Actinopterygii | Perciformes | Pinguipedidae | Parapercis | dropped^1^ |  | X | X |
| Actinopterygii | Perciformes | Serranidae | Epinephelus | dropped^1^ | X | X | X |
| Actinopterygii | Spariformes | Lethrinidae | Gymnocranius | dropped^1^ |  | X | X |
| Actinopterygii | Spariformes | Lethrinidae | Lethrinus | dropped^1^ | X |  | X |
| Chondrichthyes | Carcharhiniformes | Carcharhinidae | Carcharhinus | dropped^1^ |  | X | X |
| Actinopterygii | Spariformes | Nemipteridae | Nemipterus | dropped^1^ | X | X |  |
| Actinopterygii | Gobiiformes | Gobiidae | Navigobius | vittatus^2^ | X |  |  |
| Actinopterygii | Cypriniformes | Leuciscidae | Alburnus | alburnus^2^ | X |  |  |
| Actinopterygii | Gobiiformes | Gobiidae | Ptereleotris | grammica^2^ | X |  |  |
| Actinopterygii | Gobiiformes | Gobiidae | Ptereleotris | uroditaenia^2^ | X |  |  |
| Actinopterygii | Perciformes | Serranidae | Luzonichthys | waitei^2^ | X |  |  |
| Actinopterygii | Acanthuriformes | Acanthuridae | Naso | minor^2^ | X |  |  |
| Actinopterygii | Carangiformes | Carangidae | Atropus | atropos^2^ | X |  |  |
| Actinopterygii | Perciformes | Scorpaenidae | Scorpaenodes | xyris^2^ | X |  |  |
| Actinopterygii | Centrarchiformes | Girellidae | Girella | nebulosa^2^ | X |  |  |
| Actinopterygii | Labriformes | Labridae | Halichoeres | melanotis^2^ | X |  |  |

##

## **Table S4 |** PERMANOVA testing the effect of sampling method (i.e. Drop-Camera, BRUV or eDNA) on species richness. Significant effects are highlighted in bold.

| **Terms** | **Df** | **Sum of squares** | **Mean Sq** | **Pseudo-F** | ***p.*value** |
| --- | --- | --- | --- | --- | --- |
| Method | 2 | 7.22 | 3.61 | 15.59 | **0.001** |
| Residual | 60 | 13.89 |  |  |  |
| Total | 62 | 21.11 |  |  |  |

## **Table S5 |** Pairwise PERMANOVA comparing the effects of sampling method (i.e. Drop-Camera, BRUV or eDNA) on species richness. Significant effects are highlighted in bold.

| **Group** | **Average distance** | **t** | ***p.*value** |
| --- | --- | --- | --- |
| Drop-Camera vs BRUV | 0.83 | 4.07 | **0.001** |
| Drop-Camera vs eDNA | 0.89 | 4.08 | **0.001** |
| BRUV vs eDNA | 0.92 | 3.73 | **0.001** |

##

## References

[Belbin, L., E. Wallis, D. Hobern, and A. Zerger. 2021. The Atlas of Living Australia: History, current state and future directions. Biodiversity data journal 9:e65023.](http://paperpile.com/b/iCoGXC/bLNT)

[Larsson, A. 2014. AliView: a fast and lightweight alignment viewer and editor for large datasets. Bioinformatics 30:3276–3278.](http://paperpile.com/b/iCoGXC/Kcra)
